# Ancestral splice variation is a key substrate for rapid diversification in African cichlids

**DOI:** 10.1073/pnas.2516477123

**Published:** 2026-05-12

**Authors:** Pooja Singh, Ehsan Pashay Ahi, Anna Duenser, Marija Durdevic, Wolfgang Gessl, Sylvia Schaeffer, Julian Gallaun, Ole Seehausen, Christian Sturmbauer

**Affiliations:** ^a^Department of Biology, University of Graz, Graz 8010, Austria; ^b^Division of Aquatic Ecology and Evolution, Institute of Ecology and Evolution, University of Bern, Bern 3012, Switzerland; ^c^Department of Fish Ecology and Evolution, Swiss Federal Institute of Aquatic Science and Technology (EAWAG), Kastanienbaum 6048, Switzerland; ^d^Organismal and Evolutionary Biology Research Programme, University of Helsinki, Helsinki 00014, Finland; ^e^Federal Agency for Water Management, Institute for Water Ecology, Fisheries and Lake Research, Mondsee 5310, Austria; ^f^Diagnostic and Research Centre for Molecular BioMedicine, Institute of Pathology, Medical University of Graz, Graz 8010, Austria; ^g^Core Facility Computational Bioanalytic, Centre for Medical Research, Medical University of Graz, Graz 8010, Austria

**Keywords:** gene regulation, speciation, adaptive radiaton, alternative splicing, convergent evolution

## Abstract

Adaptive radiation is a major driver of biodiversity. In some of the largest radiations, increasing evidence suggests that explosive morphological diversification is often fueled by standing genetic variation and admixture, rather than de novo mutations. The rapid translation of this genetic variation into novel phenotypes through gene regulation remains poorly understood. Analyzing 200 transcriptomes from three independent but phylogenetically nested African cichlid adaptive radiations, we show that alternative splicing (AS) evolved faster than gene expression (GE), playing a pivotal role in shaping novel trophic adaptations in the youngest and most species rich radiations in Lakes Victoria and Malawi. This divergence was largely driven by ancestral alternative isoforms, which, though present at low levels in related lineages that did not form radiations, increased in frequency during adaptive radiation. In addition, novel isoforms of craniofacial genes—some evolving within just a few thousand years—contributed further to adaptation. The rapid turnover of AS is consistent with periods of relaxed selection followed by directional selection on alternative isoforms and splice sites, a dynamic that may have preserved a rich cache of isoform variation in these radiations and enabled ecological diversification as adaptive zones became available. We argue that the interplay between splicing and different forms of selection facilitates the generation and maintenance of protein-coding diversity, promoting evolutionary innovation into many ecologically different species at extremely short timescales.

## Significance

Adaptive radiation—when species rapidly diversify to fill new ecological niches—is a key driver of biodiversity, but its underlying molecular mechanisms remain unclear. Using comparative transcriptome sequencing across multiple African cichlid fish adaptive radiations, we found that rapid changes in alternative splicing—the process generating different protein-coding isoforms from the same gene—contributed more to early ecological divergence than shifts in gene expression levels. Most adaptive isoforms were present at low levels in the ancestor of these radiations, but some new isoforms evolved remarkably fast, shaping diverse ecologies. We show that alternative splice variation, often thought to be biological “noise,” can be a powerful and labile source of rapid evolutionary innovation during early stages of adaptive radiation.

Adaptive radiations are characterized by bursts of ecological and morphological diversification that have shaped the biodiversity on our planet (1). Rates of molecular evolution in protein-coding genes cannot explain the rate at which morphological evolution happens during adaptive radiation (2–4), which is why gene regulation is hypothesized to be an important mechanism for ecomorphological change in these lineages (5–7). Transcriptional changes in gene expression (GE) fine-tune the amount of messenger RNA (mRNA) and thus proteins produced from a gene (3, 8) and alternative splicing (AS) is a co-/posttranscriptional mechanism that can rapidly diversify the kinds of transcript and protein isoforms coded by a single gene by generating multiple distinct messenger RNAs (mRNAs) (9, 10). While some inroads have been made (7, 11–15), we still understand little about how these transcriptional and posttranscriptional regulatory mechanisms interact and are shaped by evolutionary processes to give rise to species-rich bursts of adaptive radiation (5).

**Fig. 1.**
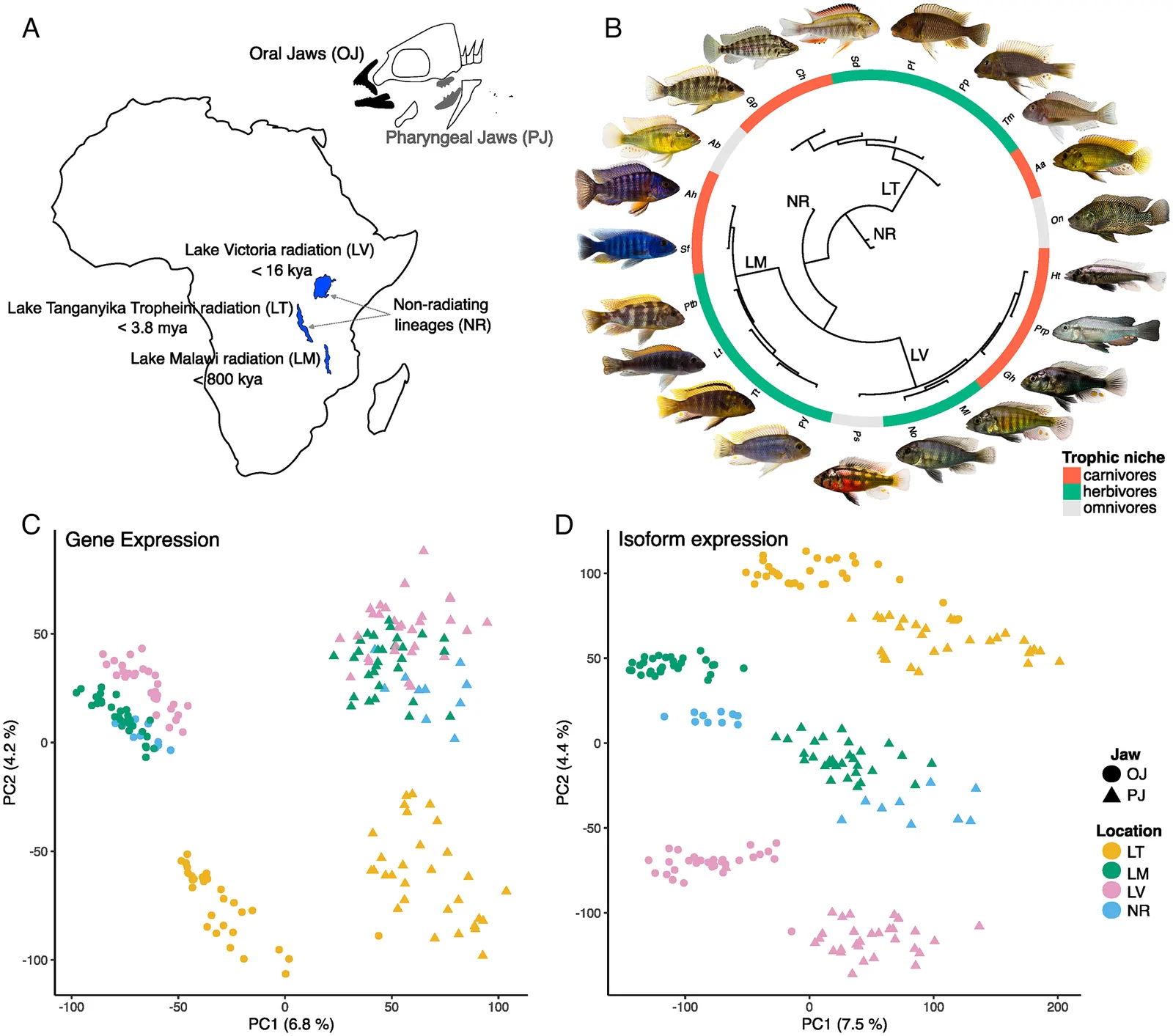
Study overview. (*A* and *B*) Biogeographical setting of haplochromine cichlid species sampled in this study (20 species, 2 jaw tissues (oral and pharyngeal), 5 biological replicates per species per tissue, n = 200). Phylogenetic relationships reconstructed using transcriptome SNPs. Lake Victoria: *Paralabidochromis sauvagei* (Ps), *Neochromis omnicaeruleus* (No), *Mbipia lutea* (Ml), *Gaurochromis hiatus* (Gh) *Prognathochromis perrieri (*Prp); *Haplochromis thereuterion* (Ht); Lake Malawi: *Aulonocara hansbaenschi* (Ah), *Sciaenochromis fryeri* (Sf), *Petrotilapia* sp. “*Thick bars*” (Ptb), *Labeotropheus trewavasae* (Lt), *Tropheops tropheops* (Tt), *Petrotilapia* sp. “yellow-chin” (Py); Lake Tanganyika: *Tropheus moorii* (Tm), *Petrochromis polyodon* (Pp)*, Petrochromis famula* (Pf), *Simochromis diagramma* (Sd), *Ctenochromis horei* (Ch), *Gnathochromis pfefferi* (Gp). Nonradiating (NR) haplochromines, *Astatotilapia burtoni* (Ab) and *Astatoreochromis alluaudi* (Aa) are included as riverine lineages that entered lakes but did not radiate, and are also phylogenetic sister lineage to the radiations. *O. niloticus* (On) is the outgroup species which was included for phylogenetic analyses only. (*C* and *D*) Principal component analysis of transcriptome-wide normalized gene and isoform expression from oral (OJ) and pharyngeal jaws (PJ) of all 20 species.

To address this knowledge gap, here we examined the evolutionary dynamics of GE and AS in cichlid fishes from Lake Victoria, Lake Malawi, and Lake Tanganyika that represent three of the fastest and ecomorphologically most diverse vertebrate adaptive radiations. Species in these lakes have evolved diverse jaws and feeding habits to adapt to different food sources, forming species-rich communities spanning complex food webs (16, 17). Across lakes, similar feeding ecologies and morphologies have evolved repeatedly, suggesting that cichlid lineages solved ecological challenges in similar ways, and that cichlid evolution may be constrained (18–23). We sequenced whole transcriptomes of oral (OJ) and pharyngeal jaws (PJ), the functional decoupling of which is considered a key evolutionary innovation that opened up novel trophic niches for cichlid fishes (24, but see 25), from 18 phylogenetically nested cichlid species. We selected species with ecologically divergent herbivorous and carnivorous trophic adaptations within lakes and ecologically convergent trophic adaptations across lakes (16, 19, 20) (Fig. 1*A*). This allowed us to address dynamics of repeated evolution as well as divergent evolution. Our sampled lineages represent the evolutionary process of adaptive radiation at different stages—Lake Victoria is the youngest and has the fastest rates of speciation, Lake Malawi is middle-aged, and Lake Tanganyika is the oldest and most ecomorphologically and genetically distinct (26, 27)—providing a powerful framework to study the temporal role of different gene regulatory mechanisms. We also included data from two nonradiating cichlid species in our study that live in these lakes, and thus have access to ecological opportunity, but did not form radiations. These nonradiating *Astatotilapia*/*Astatoreochromis* lineages are also phylogenetic sister-groups to the radiations, with whom they share a recent common ancestor (26, 28). All our selected species are from the tribe Haplochromini, which is the most species-rich and ecologically diverse cichlid group (29) (Fig. 1*B* for phylogenetic relationships). Current theory of East African cichlid dispersal suggests that an ancient riverine *Astatoreochromis*-like haplochromine lineage from Lake Tanganyika, colonized the rivers of Eastern and Central Africa, giving rise to a lineage that spread from the Congo to South Africa, and to the coast of the Mediterranean Sea (26, 28, 29). Several members of that lineage later seeded the radiations in Lake Malawi and Lake Victoria as hybrid populations (30, 31). Thus, it has been hypothesized that ancestral standing variation may have been an important driver of adaptive radiation in East African cichlids (32–34). Based on this theory, and through the sampling of lineages that formed radiations versus those that did not, we tested whether ancestral variation in GE and AS 1) contributed to divergent and convergent trophic evolution during adaptive radiation, 2) play distinct roles in biological diversification, such as rapid functional diversification during/near speciation through splicing versus much slower adaptive fine-tuning after speciation through GE, and finally 3) if we can find evidence for directional selection on alternative isoforms of craniofacial genes.

## Results

### Global Patterns of Gene and Isoform Expression.

To study the evolution of GE and AS during adaptive radiation, we generated transcriptomes of two tissues (OJ and PJ) from 100 cichlid fishes belonging to 20 species (Fig. 1 and *SI* *Appendix*, Table S1) from three lake adaptive radiations and two species that entered lakes but did not make radiations (5 individuals per tissue per species, total N = 200). Reads were mapped to the phylogenetically equidistant *Oreochromis niloticus* reference genome (35) (median sequencing depth per tissue: median ~6.4 million 125 base-pair (bp) paired end reads per library; median ~80% uniquely mapped read; *SI Appendix*, Table S1) using the genome-guided transcript assembly function. Gene annotations of species and tissues constructed using StringTie (36) were merged incrementally into one super-annotation (36) that was used for downstream analysis. This strategy helped to reduce false positives while increasing detection of novel and lowly expressed isoforms. Gene and isoform expression was quantified using established methods (36, 37).

To explore global patterns of gene and isoform expression, we performed Principal Component Analysis (PCA) across all samples for all genes. GE PC1 separated the two tissues, and on PC2, Lake Tanganyika clustered separately from the younger radiations from Lakes Malawi and Victoria and the nonradiating species, which formed an overlapping cluster (Fig. 1*C*). PCA clustering of isoform expression, which is a consequence of AS, was also tissue-specific on PC1, but clearly lake-specific on PC2 (Fig. 1*D*) with nonradiating species clustering closer to Lake Malawi samples, particularly for the PJ. Both gene and isoform expression patterns within tissues capture structure among radiations (Fig. 1*B*), with stronger signals emerging from the isoform expression. These patterns suggest that isoform-level variation may capture interspecific differences, even among very young species-flocks, that are not apparent at the GE level, although PCA provides only qualitative indication of these relationships.

### Splicing and Expression Have Contrasting Evolutionary Dynamics.

**Fig. 2.**
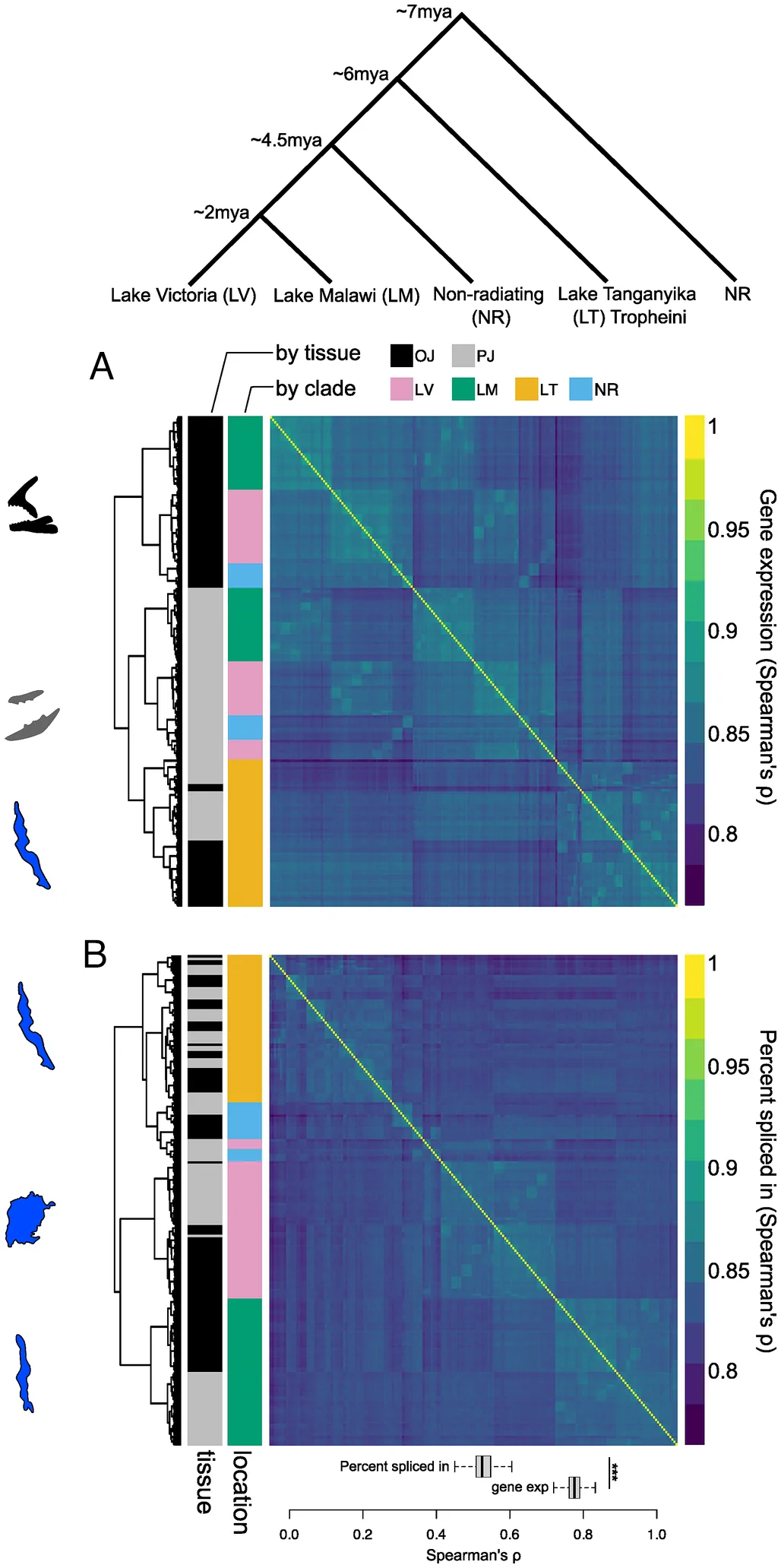
Profiling of GE and AS in East African cichlids. Hierarchical clustering of pairwise Spearman’s rank correlation coefficient (ρ) of transcriptome-wide (*A*) normalized GE (GE) and (*B*) isoform percent spliced in (PSI) of genes with more than one isoform. Analysis was conducted across all samples (n = 200). Sample clustering is represented as a tree on the *Left* side of the heatmaps, with tissue and clade (radiations or nonradiating) of origin of species annotated in colors and the dominant cluster signature illustrated as radiation/nonradiation/jaw. Boxplots show significant difference in distribution of Spearman’s ρ of PSI and GE across all samples (two-sides *t* test *P* < 0.001). See *SI* *Appendix*, Fig. S1*A* for boxplots of Spearman’s ρ of PSI and GE calculated per lake. Cladogram illustrates simplified cladogram of phylogenetic relationship of species shown in Fig. 1*B*. Divergence times taken from refs. 26 and 27. See *SI* *Appendix*, Fig. S2 for sample names.

AS results in the change of relative abundance of isoforms produced from the same gene (9), which may have evolutionary consequences (5, 38). But little is known about how AS evolves at different timescales during adaptive radiation. To address this, we calculated percent spliced in (PSI), a standard AS metric that represents the relative abundance of an isoform as a proportion of the total GE (39, 40) and computed Spearman’s rank correlation coefficients (Spearman’s ρ) across all samples and tissues for GE (all genes) and PSI (only genes with more than one isoform) to compare rate of change among these two processes. Our analysis revealed higher Spearman’s ρ values for GE than AS (Fig. 2 boxplot, two-sided *t* test *P* < 0.001). This suggests that GE was more conserved (less divergent) across samples than PSI during cichlid evolution. We next tested if PSI pattern of constitutively expressed isoforms (main isoform of a gene based on the gene annotation) were more conserved during cichlid evolution than alternative isoforms (other isoforms of a gene based on the gene annotation). Indeed, we found that Spearman’s ρ of PSI for alternative isoforms was significantly lower than that of constitutive isoforms (*SI* *Appendix*, Fig. S1, two-sided *t* test *P* < 0.001). This fits the expectation that transcriptome-wide alternative isoforms are evolving under relaxed selection pressures during adaptive radiation (41, 42).

Next, we analyzed the hierarchical clustering of Spearman’s ρ for GE and PSI. We found that GE grouped samples by tissue for younger radiations (Lakes Victoria and Malawi) and the old nonradiating species (Ab, Aa). However, for the older Lake Tanganyika Tropheini radiation, samples clustered first by clade/radiation and then by tissue within the radiation (Fig. 2*A*), defying well-established expectations of tissue-specific conservation of GE in vertebrates (43). Spearman’s ρ of PSI clustered samples first by clade/radiation, regardless of tissue, and samples from Lake Tanganyika and nonradiating species further clustered by species, regardless of tissue (Fig. 2*B* and *SI* *Appendix*, Fig. S2). The latter pattern would be expected if changes in splicing were evolving rapidly across species and clades. This suggests that evolutionary divergence in splicing exceeds tissue-specific conservation in all these radiations, and while in young radiations, this divergence is visible at the clade level, it is even visible at species-level in the oldest radiation. Hierarchical clustering relationships generally reflected phylogenetic relationships of the radiations, however, nonradiating species clustered with Lake Victoria in the GE analysis but with Lake Tanganyika in the PSI analysis. Interestingly, PJ samples of *H. thereuterion* from Lake Victoria were placed outside of the Lake Victoria clade, sister to the PJ’s of the nonradiating *A. burtoni*, which is distributed in Lake Tanganyika basin and not found in Lake Victoria. Taken together with the PCA results (Fig. 1 *A* and *B*), our results indicate that AS may have played a more important role in the evolution of clade and species-specific jaw specializations than GE. It may further suggest that heritable changes in splicing fix rapidly during/near speciation in cichlid adaptive radiations, while GE may fine-tune morphological divergence over longer evolutionary timescales.

### Genes and Isoforms Evolve Under Stabilizing Selection.

Next, we investigated the evolution of gene and isoform expression along the phylogeny (constructed using SNPs from the RNAseq data, *SI* *Appendix*, Fig. S3*A*) by fitting Brownian motion (BM) and Ornstein–Uhlenbeck (OU) processes. Ornstein–Uhlenbeck has been applied to model stochastic GE evolution toward a single optimum i.e. stabilizing selection (44). Brownian motion models neutral evolution i.e. genetic drift. The distribution of OU variance across the phylogeny, denoted “evolutionary variance” (45), depicts how constrained expression evolution is. We found that expression of most genes and isoforms was evolving under stabilizing selection (90 to 92%) and 8 to 10% of genes were evolving neutrally (chi-squared test *q*-value < 0.05, *SI* *Appendix*, Fig. S3*B* and Dataset S1). This has been previously reported for GE model species (44) but not for isoform expression. However, in contrast to GE, isoform expression had significantly higher evolutionary variance (less constraint) than GE under the OU model for both jaws (*SI* *Appendix*, Fig. S3*C*). This higher variance could be the result of weaker stabilizing selection or even strongly relaxed selection (or perhaps the result of complex mix of selection) acting on alternative isoform expression than constitutive isoform expression, the latter being under stabilizing selection to preserve gene function. These results reinforce the idea of fast evolving AS during adaptive radiation.

### Most Splice Variation Is Ancestral, but Some May Be Novel.

**Fig. 3.**
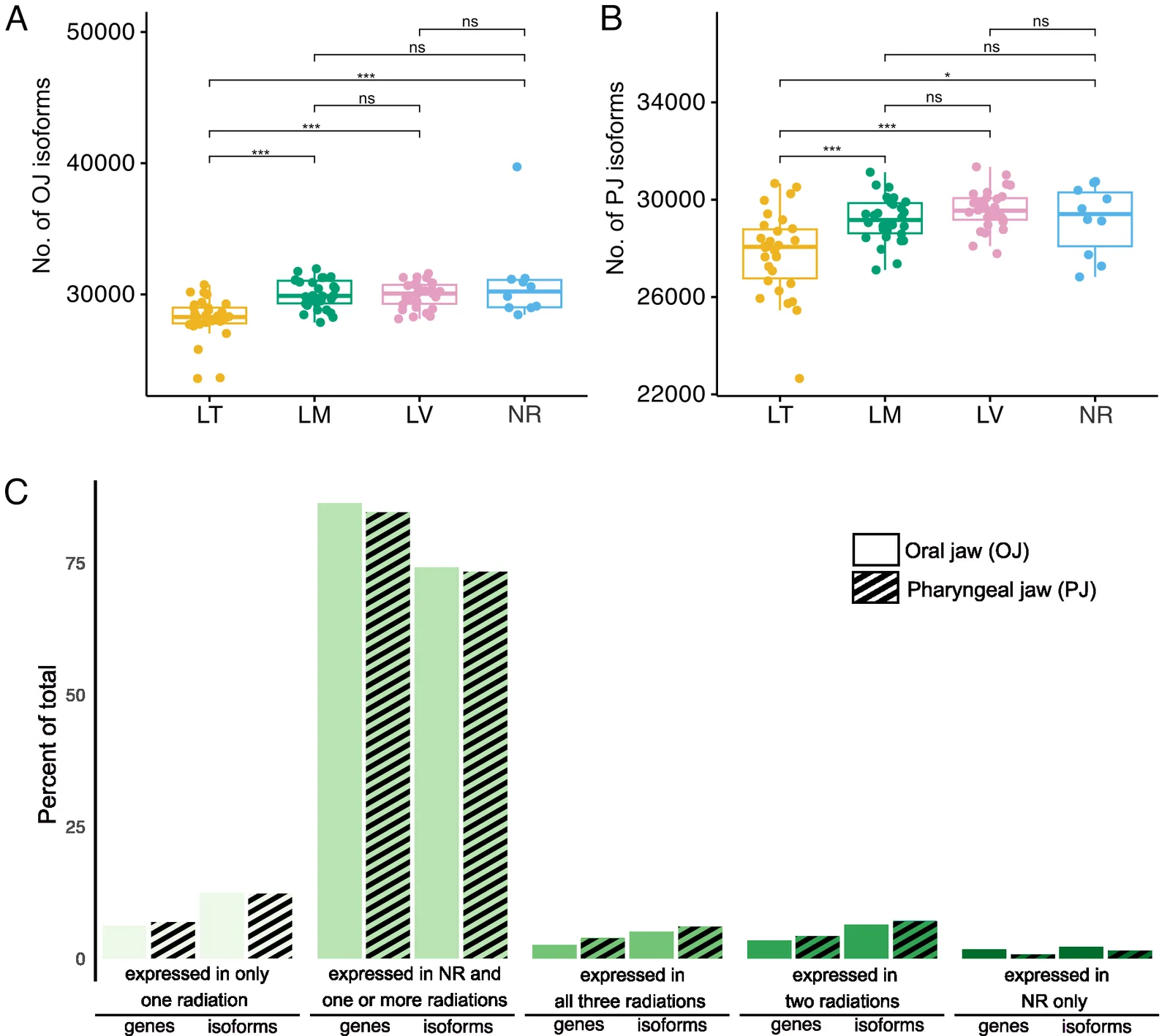
Transcriptomic variation in nonradiating and radiating cichlids. (*A* and *B*) The absolute number of expressed isoforms identified in the oral and pharyngeal jaw mRNA of all samples. The absolute number of genes expressed is shown in *SI* *Appendix*, Fig. S4. Asterisks denote Mann–Whitney *U* test significance: ***P < 0.001, **P < 0.01, *P < 0.05. NS: no significance (*C*) Percent of total genes and isoforms expressed in nonradiating species and radiations or unique to one or more of the radiations for both jaws. LV: Lake Victoria, LM: Lake Malawi, LT: Lake Tanganyika, NR: nonradiating.

Ancestral standing variation is proposed to fuel rapid cichlid adaptive radiations (34). One way that this could happen is through the sorting of ancestral variation in gene regulation (46), such as splice variation. As a first step to test if ancestral splice variation contributed to trophic divergence, we quantified the number of genes and isoforms expressed uniquely in each radiation versus those shared between radiations and nonradiating species. The total number of both genes and isoforms expressed in the OJ was higher than in the PJ; and regardless of tissue, the absolute number of genes expressed were significantly higher in samples from the younger radiations (Lakes Malawi and Victoria) and the nonradiating lineages than the older radiation of Lake Tanganyika (Mann–Whitney *U* test *P* < 0.001; Fig. 3 *A* and *B* and *SI* *Appendix*, Fig. S4 *A* and *B*). This overall pattern was also observed when we analyzed the number of isoforms per gene (*SI* *Appendix*, Fig. S5) but the difference was only significant between the PJ’s of Lake Victoria and Malawi (Mann–Whitney *U* test *P* < 0.05). We tested the homogeneity of variance among groups using the Barlett’s test and found significant differences for the number of isoforms per gene for PJ (*P* = 0.017) but not the OJ. This suggests that while the absolute number of isoforms expressed per gene is reduced over time, the transcriptional variance among species increases with time since the onset of adaptive radiation. To understand how much of this expression variation was ancestral versus putatively de novo, we investigated if each gene or isoform was expressed in only one of the clades, or across several radiating and nonradiating clades (Fig. 3*C*). The majority of genes (86.0%) and isoforms (73.0%) were expressed in at least one nonradiating species and one or more radiations—this variation is likely ancestral to all East African haplochromines. These results are consistent with what we understand about the dispersal of haplochromine cichlids that may have arisen in LT (28) and subsequently spread across much of Africa through rivers (26, 27, 29, 47). Descendants of these riverine haplochromines lineages much later colonised LM and LV (30, 31). The colonizers to LV came from the Congo and the Nile (31), those to LM came from Central and Eastern Tanzania (30). Thus, all three cichlid radiations of LT, LM, and LV would presumably share some ancestral variation, which would certainly impact the transcriptional landscape within each radiation. Furthermore, demographic history of these radiations probably also plays a role, as Lake Victoria has dried up and refilled (most recently 16,000 years ago) and this would impact the shared gene regulatory variation across all three lakes. All these factors probably contribute to why so little gene/isoform expression is shared across all three radiations, but larger sharing is observed in different subsets of radiating/nonradiating species. Given what we know about their phylogenetic relationships (Fig. 1*B*), the most parsimonious explanation is that this variation was ancestral and was lost in some radiations; instead of being independently gained in nonradiating lineages and a subset of radiations. A small subset of isoforms (~12.0%) were unique to individual radiations—pointing toward the evolution of novel splice variation in the direct ancestor of each adaptive radiation—or less likely, repeated loss in all other lineages. Novel isoforms can rapidly evolve through the weakening of purifying selection on alternative splice sites (42). It is of course possible that these putatively novel isoforms may be ancestral but were missed by our sample selection. It is also possible that isoform variation arrived through hybridization, as cycles of hybridization have fueled cichlid adaptive radiations (26, 27, 30, 31). Taken together, our data support the hypothesis that substantial transcriptomic variation existed in the gene pool of the common ancestor of all East African haplochromines that was likely shaped by selection and drift during adaptive radiation, with some isoforms being purged and others retained; with some putatively novel isoforms evolving in the ancestors of the individual radiations or in the process of adaptive radiation itself. But did these ancestral and novel isoforms contribute to ecological adaptation in cichlid radiations?

### Trophic Adaptation From Old and New Splice Variants.

**Fig. 4.**
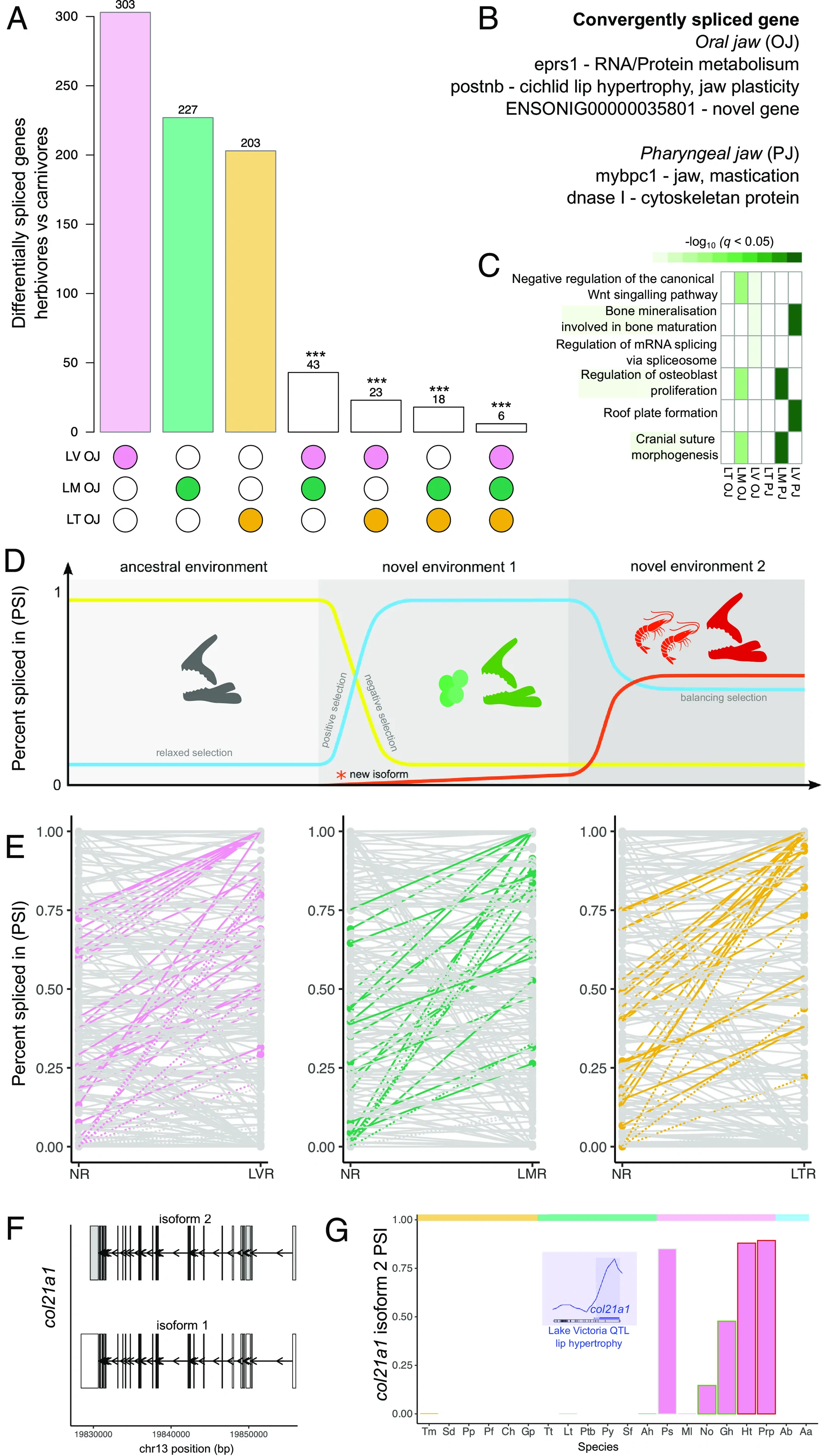
Evolutionary dynamics of differentially spliced genes between herbivores and carnivores species. (*A*) Number of significantly differentially spliced genes between oral jaws (OJ) of carnivores and herbivores within each lake radiation (*q* < 0.05) with overlaps shown (hypergeometric test *P* < 0.001). The number of differentially spliced genes between the pharyngeal jaws (PJ) of carnivores and herbivores within each radiation is shown in *SI* *Appendix*, Fig. S6*A*. (*B*) Genes that were convergently spliced in at least two radiations in the same direction (higher in herbivores or higher in carnivores). No genes were convergently spliced in the same directly in all three radiations. (*C*) Gene Ontology enrichment of differentially spliced genes between herbivores and carnivores. Only a subset of terms are shown. The full dataset is presented in *SI* *Appendix*, Fig. S7. (*D*) Hypothetical expectations for isoform frequency (measured as percent spliced in PSI) during ecological adaptation. Figure adapted from Singh and Ahi (5). (*E*) Testing the hypothesis depicted in (*D*) in our transcriptome data from Lake Victoria radiation (LV), Lake Malawi radiation (LM), and Lake Tanganyika radiation (LT) compared to nonradiating species (NR). Mean PSI was calculated all individuals of all species in each group (NR, LV, LM, LT). All lines represent all isoforms of genes that were significantly differentially spliced between herbivores and carnivores within each radiation. Solid gray lines represent all isoforms that were expressed at lower or similar levels in the nonradiating species and species from each radiation (PSI differences <= 0.2). Dotted gray lines represent all isoforms that were not expressed in nonradiating species but were expressed species from each radiation (PSI differences <= 0.2). Colored solid lines indicate isoforms that were expressed at low levels in nonradiating species but were higher expressed in radiating species (PSI differences > 0.2). Colored dotted lines indicate isoforms that were not expressed in nonradiating species but were expressed in radiating species (PSI differences > 0.2). (*F*) The two isoform models for *col21a1* in the gene annotation. (*G*) Species mean expression of *col21a1* isoform 2 across all species in our dataset. Inset highlights a known QTL lip shape from Lake Victoria containing *col21a1*.

Since the global patterns of AS revealed that it was evolving rapidly in the jaws of cichlids during adaptive radiation and that much of the splice variation was ancestral, we next asked if ancestral splice variants contributed to trophic divergence of cichlid jaws. We conducted differential PSI analysis between the jaws of species adapted to herbivorous and carnivorous diets (see Fig. 1 for trophic specialization of species) within each lake to identify differentially spliced genes (DSGs) that may play a role in shaping their eco-morphological differences. The largest number of genes were differentially spliced in Lake Victoria (OJ 303, PJ 215) followed by Lake Malawi (OJ 227, PJ 214) and Lake Tanganyika (OJ 203, PJ 126) (Fig. 3*A* for OJ, *SI* *Appendix*, Fig. S6 for PJ, Dataset S1). This indicates that younger cichlid radiations leveraged splice variation relatively more than the older radiation in Lake Tanganyika for trophic adaptation. Differentially spliced genes were significantly enriched (Fisher’s exact test *q* < 0.05) for GO terms specifically associated with craniofacial and jaw morphogenesis that are known from literature such as “regulation of osteoblasts,” “bone mineralization involved in bone maturation,” “roof plate formation,” and “skeletal muscle development” (subset shown in Fig. 3*C*; full data in *SI* *Appendix*, Fig. S7). This was especially true for DSGs in the younger radiations. The Wnt signaling pathway that has been associated with the evolution of new craniofacial phenotypes at the larval stage in Malawi cichlids (48) was enriched in divergent jaws from Lake Victoria. It is likely that AS of Wnt pathway genes *gpc3* and *lzts2a* (Dataset S1) that are known to control skeletal development and dorsoventral patterning (49, 50), contributed to adaptive jaw shape differences among Lake Victoria cichlids. Two genes, *rnfox1* and *rbm5*, involved in “mRNA splicing via the spliceosome” were differentially spliced in Lake Victoria oral jaws (Dataset S1). This suggests that AS of spliceosome components, which is the molecular machinery that controls splicing, contributed to the evolution of ecological flexibility in the fastest and most species-rich vertebrate adaptive radiation. In Lake Malawi OJ, differential splicing of genes involved in bone maturation (*col1a1*) and cranial morphogenesis (*sp7*) were notable. Overall, twice as many DSGs were shared between Lakes Victoria and Malawi, than either of these lakes shared with Lake Tanganyika (Fig. 4*A* and *SI* *Appendix*, Fig. S6), which is most consistent with these splice variants evolving and/or gaining function in the ancestor of these radiations.

Of the genes that were significantly differentially spliced between herbivores and carnivores in each lake (Fig. 3*A* and *SI* *Appendix*, Fig. S6*A*), a dynamic pattern of isoform evolution emerged: Many of these were low expressed isoforms in nonradiating species that increased in expression in radiating species (Fig. 3*E* for OJ, *SI* *Appendix*, Fig. S6*B* for PJ). An increase in frequency would be the expectation if these low expressed ancestral alternative isoforms persisted in populations as standing variation due to relaxed selection pressures (41, 42) and gained adaptive function when these lineages entered lakes and discovered newly available food sources (Fig. 3*D*) as hypothesized by Singh and Ahi (5). Many highly expressed isoforms in nonradiating species also decreased in frequency or were lost in the lacustrine radiations (presumably as the alternative isoform increased in frequency). We further identified 34 potentially novel isoforms that were absent in the two sampled nonradiating species and only expressed in a subset of the three adaptive radiations (Fig. 3*E* dotted lines, Dataset S1). Sixteen of these 34 novel isoforms were found in Lake Victoria, followed by 12 in Lake Malawi and six in Lake Tanganyika. Novel isoforms of note were of genes *col21a1* and *lzts2a*. *Col21a1* had two isoforms (Fig. 3*F*) of which the main isoform is expressed in the OJ of Lake Victoria herbivores as well as herbivores from Lakes Tanganyika and Malawi (with one exception, Dataset S1). The alternative isoform of *col21a1* is only expressed in Lake Victoria species, with higher expression in carnivores and omnivores (Fig. 3*G*). *Col21a1* is associated with human cleft lip syndrome and maps to a QTL for hypertrophic lips in Lake Victoria cichlids (51). It is conceivable that this isoform may have arisen de novo in Lake Victoria cichlids or arrived from one of the riverine haplochromines that colonized Lake Victoria and hybridized (52). In the future, integrating transcriptomic analysis with population genetics and functional genomics (F_ST_ scans, GWAS, spliceQTL) approaches would help to narrow down the genetic variants associated with splicing and the evolutionary processes that shape them. Our results also highlight the conservation of craniofacial development pathways across vertebrates, and the promise of cichlid fishes as a natural model for studying human craniofacial disease (53).

### Differential Expression of Major Craniofacial Genes Contributed to Jaw Divergence.

**Fig. 5.**
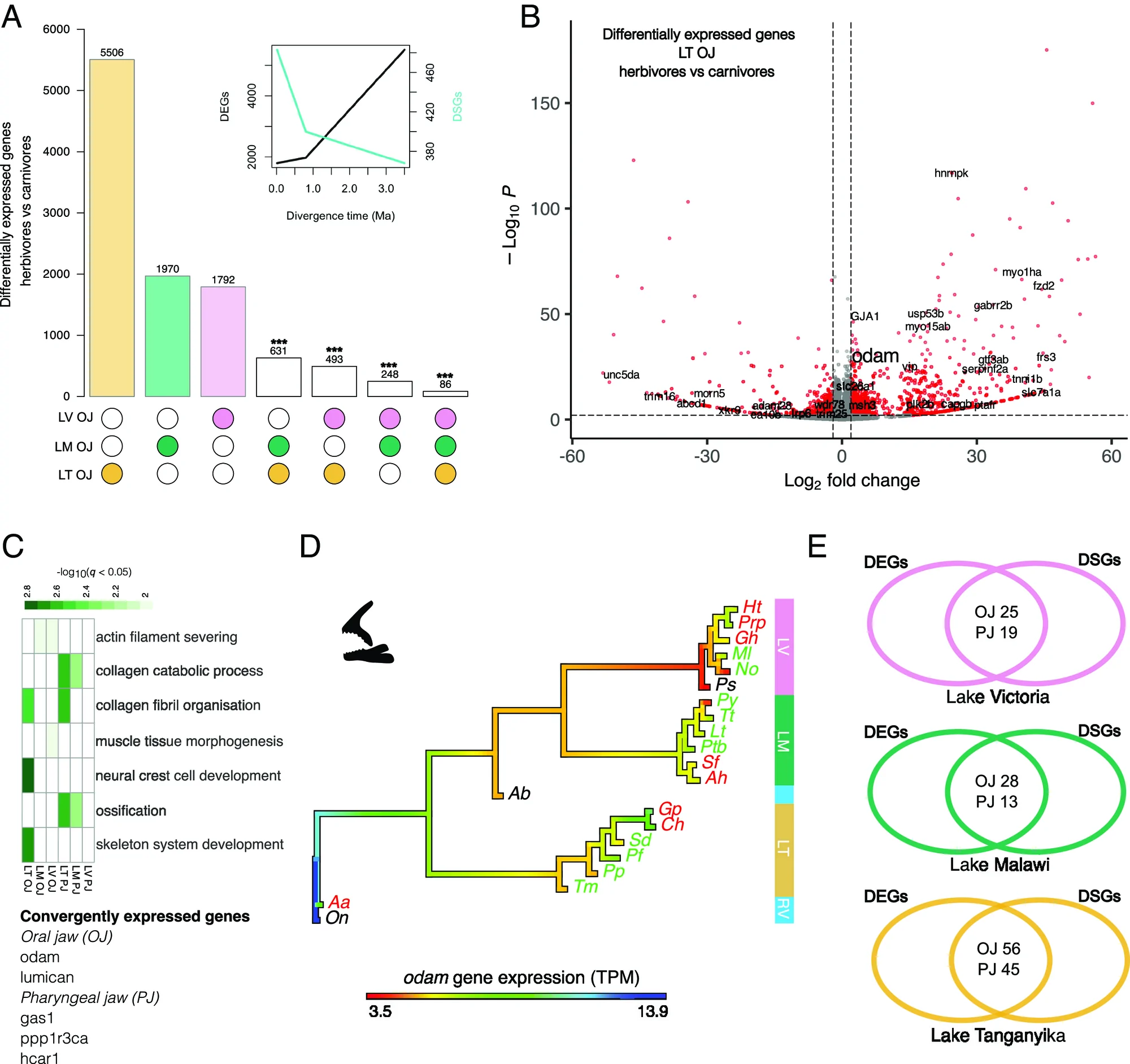
GE divergence in oral jaws of herbivore and carnivore species. (*A*) Number of differentially expressed genes (DEGs, *q* < 0.05) between oral jaws (OJ) of herbivores and carnivores in each lake, with overlaps annotated (hypergeometric test *P* < 0.001). Results for pharyngeal jaws can be found in *SI* *Appendix*, Fig. S8. The inset shows the relationships between number of DEGs and differentially spliced genes (DSGs) in each cichlid radiation plotted as function of divergence time. (*B*) Volcano plot illustrating DEGs in Lake Tanganyika (LT) oral jaws. Significant DEGs are highlighted in red. *Top* gene outliers are annotated. (*C*) Gene Ontology enrichment of DEGs between herbivores and carnivores. Only a subset of terms are shown that are known in literature to be associated with jaw/craniofacial divergence. The full list is presented in *SI* *Appendix*, Fig. S9. Some candidate genes for jaw morphogenesis/craniofacial development that were convergently expressed in all three radiations in the same direction (higher in herbivores or higher in carnivores). See Dataset S1 for the full list. (*D*) Ancestral state reconstruction of GE of the convergently expressed *odam* gene. (*E*) Overlap of DEGs and DSGs between herbivores and carnivores within each lake radiation.

In addition to differences in splicing, we investigated GE differences between herbivores and carnivores in each lake radiation to be able to compare the role of GE with AS in trophic adaptation. The largest number of differentially expressed genes (DEGs) were in Lake Tanganyika (OJ 5506, PJ 4094), followed by Lake Malawi (OJ 1970, PJ 1499) and Lake Victoria (OJ 1792, PJ 1166) (Fig. 5*A* for OJ, *SI* *Appendix*, Fig. S8 for PJ, Dataset S1). Several interesting candidate genes for jaw/craniofacial morphogenesis known from literature stood out as the most extreme outliers in the oldest Lake Tanganyika where GO terms such as “neural crest cell development,” “skeletal system development,” and “ossification” were significantly enriched (*q* < 0.05). One such gene for Lake Tanganyika OJ was Frizzled 2 (*fzd2*), a Wnt pathway gene that is highly expressed in the developing face of vertebrates, and mutations of this gene cause cleft palate and wider nose and upper jaw (54). A gene controlling teeth morphology in cichlids, *odam* (55), and a gene linked to craniofacial disease, *gja1,* were also significant for Lake Tanganyika OJ (56). Two interesting outliers in Lake Victoria OJ were *kaznb*, a craniofacial development gene that has been found to be upregulated in the jaws of herbivorous cichlids at larval and adult life stages (57) and *egr1*, that forms part of the regulatory cascade modulating cranial bone morphogenesis through the BMP pathway (58). Interestingly, one of the most extreme outliers for Lake Tanganyika OJ was *hnRNPk*, an RNA binding protein that regulates pre-mRNA splicing (59). Overall, the GO terms for DEGs in the youngest lakes were less specific to jaw development than the DSGs. This suggests that shifts in AS may be a more flexible route to rapid diversification in explosive adaptive radiations than changes in GE.

### Contrasting Functions for AS and GE.

We found dissimilar patterns in the extent of differential GE and AS underlying divergent trophic adaptation across cichlid radiations. Even though the overall number of DEGs was much higher than DSGs across all three radiations (Figs. 4*A* and 5*A* and *SI* *Appendix*, Figs. S6 and S8), the relative number of DEGs across radiations was the highest in the oldest radiation and lowest in the youngest radiation, while the DSGs showed the opposite pattern (Fig. 5*A*, *Inset*). Interestingly, more DEGs in each of the younger radiations overlapped with the older radiation, in contrast more DSGs overlapped among the younger radiations (Figs. 4*A* and 5*A* and *SI* *Appendix*, Figs. S6*A* and S8). This pattern indicates that a complex temporal interplay of GE and AS underlies adaptive radiation, with ancestral splice variation enabling rapid ecological diversification at early stages of speciation, while GE differences evolve much slower, most likely fine-tuning morphological adaptations long after speciation. This would be consistent with the GO enrichment results for differentially spliced and expressed genes that we found in younger versus the older radiation.

It has been proposed that AS and GE perform contrasting biological functions (11, 57, 60–62). However, these studies have lacked a phylogenetic perspective. In our dataset, the overlap of genes that were both differentially spliced and differentially expressed between herbivores and carnivores followed a phylogenetic and evolutionary pattern. The highest overlap was in Lake Tanganyika (OJ 56, PJ 45), followed by Lake Malawi (OJ 28, PJ 13) and Lake Victoria (OJ 25, PJ 19) (Fig. 5). Our results reinforce the idea that putatively adaptive AS and GE changes act mostly on different genes—but we show that the extent of gene overlap depends on the evolutionary age of lineages, with fewer genes being regulated by both processes in more recently diverged species.

### Convergent GE and Splicing Underlying Convergent Trophic Evolution.

African cichlid radiations are an ideal system to study the repeatability of evolution as they have evolved convergent trophic adaptations, particularly in jaw shape, to exploit similar food sources in similar ways in different lakes (19). See results of geometric morphometric analysis in the Supplementary material that illustrates that herbivores and carnivores cluster together in a jaw shape PCA regardless of which lake they are from; and that jaw shape is diverging along parallel axes in the three radiations. We asked if differentially expressed and spliced genes contributing to herbivory vs carnivory showed parallel divergence across all three radiations. We find convergent DEGs in both oral (23 upregulated, 12 downregulated in herbivores) and pharyngeal jaws (14 upregulated, 6 downregulated in herbivores) that fit this pattern (Fig. 5 and *SI* *Appendix*, Fig. S8). Several convergently used OJ genes have been associated with the development of teeth (*odam*), bone (*lum*), and muscle (*myrip*, *myo15aa*) (Fig. 5 *B* and *C* and Dataset S1). *Odam* expression has been associated with larger vs smaller teeth in cichlid fishes and our ancestral state reconstruction suggests that this gene increased in expression repeatedly in the oral jaws of carnivores in each lake radiation (Fig. 5*D*). Convergent genes for PJ were *hcar1*, *ppp1r3c*, myl13, and *gas1*. *Gas1* interacts with *shh* in early face development (63). We validated the convergent expression of *odam, gas1, hcar1,* and *ppp1r3ca* using qPCR (*SI* *Appendix*, Fig. S10). No DSGs were convergently spliced in the same direction in all three radiations. We only found evidence of convergent splicing in the same direction in two of the three radiations in the following noteworthy genes: OJ: *eprs1*, *postnb* and PJ: *mybpc1*, *Dnase1* (Fig. 4 *A* and *B*). These results suggest that jaw adaptation in cichlids is a polygenic trait with different genes functioning redundantly across these three radiations. The redundancy is higher in the regulation of genes through AS than GE, most likely due to the species-specific nature of AS.

## Discussion

Bursts of diversification that characterize adaptive radiations have challenged the expectations of gradual evolution (64, 65). In these lineages, rapid morphological divergence is often fueled by admixture and standing genetic variation rather than the slow accumulation of protein-coding mutations (66, 67). However, we still understand little about how this genetic variation shapes adaptive radiations through different gene regulatory mechanisms (5, 11, 68). Through the analysis of 200 transcriptomes representing three independent but phylogenetically nested cichlid adaptive radiations that evolved between <16,000 to 3.8 million years ago, we investigated the landscape of GE and AS underlying trophic adaptation. We show that the ancestors of East African haplochromine cichlids harboured a rich cache of isoform variation generated by AS. This isoform variation evolved faster than overall GE to rapidly give rise to novel trophic adaptations when ecological opportunity arose. This pattern was most pronounced in the youngest and most species rich adaptive radiations in Lakes Victoria and Malawi that had relatively higher differences in splicing than Lake Tanganyika-Tropheini for genes associated with jaw morphogenesis. We further found that novel isoforms that most likely evolved in the course of adaptive radiation also contributed to adaptation. The rapid turnover of AS that we observed here is consistent with relaxed selection pressures on splice-sites, as alternative isoforms can escape the effects of purifying selection as long as the major isoform is expressed (41). Theory suggests that relaxed selection can increase the rate of evolution of complex adaptations in heterogenous environments, allowing populations to rapidly cross adaptive valleys (69). We propose that relaxed selection maintaining standing splice variation, followed by periods of positive selection on alternative isoforms of craniofacial genes contributed to the rapid evolution of trophic adaptations in cichlids as they entered lakes and were exposed to new and ecologically more stable environments. An alternative explanation for our observation can be the complex influence of diverse forms of selection (perhaps in combination with neutral processes), such as balancing selection, frequency-dependent selection, and diversifying selection, that promote isoform diversity. Taken together, our findings challenge the long-held notion that most alternative isoforms are evolutionary noise (70), because this noise can fuel innovation when ecological opportunity arises, providing one possible mechanistic explanation for how these extraordinary cichlid radiations were able to exploit ecological opportunity as such extremely short timescales.

Our results on the evolutionary dynamics of GE are partially consistent with previous work on transcriptome evolution at deeper timescales. In contrast to AS, GE is largely conserved at the tissue-level during vertebrate evolution (43, 44, 68, 71). Remarkably, we found lineage-specific clustering of GE in the jaws of Lake Tanganyika-Tropheini (Fig. 2). Tropheini are named after their diverse trophic adaptations that allowed them to enter the littoral zones of Lake Tanganyika at a much later stage and successfully diversify alongside other established cichlid radiations (27, 72, 73). They were able to do this by rapidly remodeling their jaw, dentition, and craniofacial morphology to forage foods in new and different ways (72). Our findings may reflect the particular importance of GE regulation for evolutionary transitions in Tropheini jaws during speciation (11).

We find that signatures of splicing became increasingly more species-specific, and GE became more lineage-specific over evolutionary time (Fig. 2). However, species-specific signatures of splicing do not emerge until ~3.8 million years of evolution, a pattern not observed in previous large-scale studies on older lineages (43, 71). We also found that both rapidly evolving AS and GE contributed to jaw adaptations in herbivorous and carnivorous cichlids, but their contribution was inversely predicted by evolutionary age (Fig. 5*A*). Relatively more differences in AS were found to underlie trophic differences in jaws of cichlids from the younger radiations, while GE differences dominated in the older radiation. This was also reflected in the biological functions of these genes: 1) more jaw morphogenesis related gene ontology terms were enriched in differentially spliced genes in Lakes Victoria and Malawi than Tanganyika; 2) in Tanganyika more jaw morphogenesis related gene ontology terms were enriched in differentially expressed genes. This pattern indicates that a complex temporal interplay of GE and AS underlies adaptive radiation, with ancestral splice variation enabling rapid ecological diversification at early stages of speciation, while GE differences evolve much slower, mostly likely fine-tuning the adaptive morphology long after speciation. Consistent with previous studies, we found many genes implicated in jaw divergence suggesting that jaw remodeling is a complex phenotype governed by myriad genes (15, 19, 27, 74). The Wnt pathway, which is a key player in the evolution of new jaw phenotypes at early stages of development (48), emerged an important candidate for jaw adaptation in our study with genes regulated through splicing (*gpc3*, *lzts2a*) and differential expression (*fzd2*). Overall, more convergent differences in GE than AS were found to underlie convergent trophic jaw adaptations across all three cichlid radiations (Fig. 4 *A* and 5*A*). The lower phylogenetic convergence of AS may underscore its role in shaping functional genetic redundancy—where multiple genetic pathways can produce similar phenotypic outcomes (75). In our case, it is not isoform diversity within a single gene that underpins redundancy (76) but rather the use of different genes regulated by lineage-specific splicing patterns, highlighting a novel form of genetic redundancy mediated by AS. Such redundancy may allow different lineages to evolve comparable traits via distinct molecular routes, increasing evolutionary flexibility and speed.

It has been hypothesized that ancestral and admixture variation plays an important role in the rapid adaptive capacity of African cichlids (34, 66). But, little empirical evidence exists explaining how this ancestral variation mechanistically plays out into morphological diversity. We show that ancestral splice variants that were differentially spliced between jaws of herbivores and carnivores are lowly expressed in nonradiating species but increased in frequency in radiating cichlids, most likely enabling trophic divergence. First, this suggests that old genetic variation can fuel new adaptations (31, 34); and that the ancestors of the East African cichlid adaptive radiations, which were most likely widely dispersing riverine cichlids, acted as reservoirs of genetic variation for diversification on a continental scale (47). And second, even if most ancestral alternative isoform variants are nonfunctional (70), lowly expressed, and evolving stochastically (41), they represent a cache of standing variation for adaptive evolution when ecological opportunity arises (5, 77). Standing variation in AS has been shown to be important for the rapid domestication of sunflowers (77), which represents one ecological transition, while cichlid adaptive radiations involve multiple, often convergent, diverse ecological transitions and speciation events. In addition to ancestral variation, we found evidence of potentially novel isoforms contributing to adaptive jaw differences in East African cichlids. This emphasizes how fast new protein-coding variation can be generated for evolution through AS.

Our present study is one of the most comprehensive cross-lake analysis of transcriptomic variation in cichlid fishes to date. However, it still represents a small subset of the hundreds of cichlid species that are found in the African Great Lakes. In the future, expanding the analyses to include more species would add more confidence to our findings on convergent and divergent GE and AS patterns underlying trophic evolution in these radiations. Furthermore, including more cichlid species that entered lakes and had access to ecological opportunity but did not form radiations (78), or riverine cichlids that are hypothesized to have contributed genetically to the lake radiations through hybridization (26, 30, 52), would shed light on how different evolutionary processes promote (or constrain) rapid diversification by rewiring transcriptional variation. Targeting a single, albeit ecologically and morphologically important, developmental stage is another constraint of our approach. In the future, studying gene regulation across multiple developmental stages (see 57) and different ecologically important tissues, would further enhance our understanding of gene regulation and the origins of diversity.

Taken together, our findings suggest that AS may be one piece of the puzzle explaining how ecological and species richness can evolve from standing genetic variation when sufficient time is not available for new protein-coding mutations to arise, fix, and be replenished. Future studies on gene regulation in evolutionary biology would benefit from moving away from a GE-centric view to a more holistic model that incorporates other levels of gene regulation, such as splicing.

## Materials and Methods

### Study Design and Sampling.

In this study, we focused on six cichlid species from Lake Tanganyika, six cichlid species from Lake Malawi, six cichlid species from Lake Victoria as well as two cichlid species from surrounding rivers, all of which are part of the “modern haplochromine” adaptive radiations (Fig. 1). The 18 species from the three lakes represent eco-morphological replicates of herbivore and carnivore trophic adaptations. We focused on the functionally decoupled oral (OJ) and pharyngeal jaw (PJ) tissues, that are involved in food capture and processing.

Samples were collected either through aquarium trade or fieldwork within the framework of collaborations with Zambian and Tanzanian fisheries (TAFIRI). All fish were reared in the fish facility at University of Graz under standardized conditions regarding tank environment, diet, and water parameters to minimize plastic effects. Fertilized eggs were taken from the mouth of mouthbrooding females 24 h after fertilization, by applying slight pressure onto their cheeks. The eggs were reared in a separate tank until the yolk sac was completely absorbed, which is also known as the developmental stage 26—end of larval stage and beginning of juvenile stage, when bony elements have formed but the organism is still growing actively (79–81). Late larval and juvenile developmental stages are ecomorphologically informative stages that have been used to study jaw adaptation and divergence cichlids (11, 82, 83). The external morphology and internal bone and cartilage development at stage 26 is illustrated in *SI* *Appendix*, Fig. S11. Stage 26 larvae were ethically sacrificed and stored in RNAlater in a fridge for seven days and then moved to a -80 °C freezer (11, 15, 76).

### RNA Extraction.

Each larva was dissected under a stereo microscope to dissect the oral jaws (OJ: bone, teeth) and the pharyngeal jaws (PJ: bones and teeth) separately (*SI* *Appendix*, Fig. S12). The bones were cleaned as much as possible, but some surrounding tissue (i.e. skin, cartilage, muscle) remained, especially for the OJ, as small size of the larvae (<1 cm) makes the dissection challenging. The dissected tissue was submerged in a lysis buffer and 1-thioglycerol according to the protocol of the Promega RNA Reliaprep kit. The sample in the lysis buffer was crushed with a ceramic bullet in a homogenizer (FastPrep-24, MP Biomedicals, Santa Ana, CA) before further processing. Two individuals were pooled per sample to increase the amount of RNA for Lake Victoria species. For Lake Malawi and Tanganyika, one individual yielded sufficient RNA. RNA quality was tested at a TapeStation 2200 with RNA Screen Tapes. It was tried to reach a RINe number of >= 7 for each sample. We extracted RNA for 5 biological replicates per jaw per species, which resulted in 200 mRNA samples in total.

### Library Preparation and Sequencing.

Library preparation was done with the TruSeq Stranded mRNA Sample Prep Kit using 24 indexing adapters and following the standard protocol. For the input RNA, we tried to reach 1,000 ng for each sample. The quality of the cDNA was tested on a TapeStation 2200 with D1000 Screen Tapes. Sequencing was executed at the Vienna BioCenter Core Facilities on a HiSeq 2500 with paired end 125 cycles per read (2 x 125 cycles). Demultiplexing was conducted by the same facility.

### Transcriptome Assembly.

Each sample had approximately ten million paired-end reads. After a quality check with Fastqc (v0.11.8) (84) and a trimming step with Trimmomatic (v0.3.9) (85), only reads with a phred > 28 and a minimum length of 70 bp were retained. To enable the assembly of novel isoforms we developed a bioinformatics pipeline with the following steps: Reads were assembled using STAR (v2.7.3.a) (86) in reference guided mode using the *Oreochromis niloticus* reference genome (35). This genome was selected because it is the best assembled and annotated cichlid reference genome and is phylogenetically equidistant to the species sampled in this study. Mapping statistics were generated with samtools idxstats (v1.9) (36, 87). StringTie (v2.0.6) was run first both with a reference genome to assemble the RNA-Seq alignments into potential transcripts and in genome-guided mode. This was done separately for the single files (per individual). The single gtf files for each biological replicate were then incrementally merged according to species and tissue and finally into one super-annotation. These repeated merging steps were conducted to reduce the probability of false positives in isoform assembly and accurately identify novel species-specific or lower expressed isoforms. To estimate the accuracy of the produced annotation files we compared them with gffcompare (v0.11.2) (88) to our reference *O. niloticus* annotation. We filtered for monoexonic transcripts not contained in the reference and a class code assigned by gffcompare (v0.11.2), indicating “possible polymerase run-on” fragments. The maximum intron length was reduced to 200,000 bp, which is the maximum intron length found in the *O.niloticus* reference. Based on the super-annotation, the expression estimates were generated with StringTie allowing no multimapping. From these expression estimates, count matrices were produced using a perl script from the griffith lab (https://rnabio.org) to extract raw count data from StringTie results. The code used for our RNAseq pipeline is on GitHub (89). SUPPA2 (v2.3) (40) was used to calculate percent spliced in (PSI) index for each isoform for each gene.

### Differential GE Analysis.

We used DESeq2(34)in R(89) to detect DEGs running comparisons 1) between herbivores and carnivores within each lake radiation for each tissue separately and 2) between oral and pharyngeal jaws within each radiation. DESeq2 uses raw read counts and estimates variance-mean dependence based on a model that utilizes the negative binomial distribution. The cutoff for DEGs was chosen at a false discovery rate of (*q* < 0.05).

### AS Analysis.

AS analysis was conducted with SUPPA2 (v2.3) (40) to evaluate differential transcript usage (DTU) between herbivores and carnivores within each lake radiation for each tissue separately. Visualization of PSI analysis was conducted in R (90).

### Modeling Gene and Isoform Expression.

We used the methods proposed by Chen et al. (45) to model gene and isoform expression along the phylogeny using Ornstein–Uhlenbeck (OU) and Brownian Motion (BM) processes. The OU process is a modification of a random walk, describing the change in expression (dX_t_) across time (dt) by dX_t_ = σdB_t_ + α (θ – X_t_) dt, where dB_t_ denotes a Brownian motion process. The model elegantly quantifies the contribution of both drift and selective pressure for any given gene: 1) Drift is modeled by Brownian motion with a rate σ while 2) the strength of selective pressure driving expression back to an optimal expression level θ is parameterized by α. The OU process accounts for phylogenetic relationships, thus allowing us to fit individual evolutionary expression trajectories.

### Phylogenetic Analysis and Ancestral State Reconstruction of GE.

We constructed expression trees using the neighbor-joining approach based on pairwise distance matrices between samples using data from all genes and applying functions from the “ape” package (v5.8.1) (91) in R (90). Distances were computed as 1 − ρ, where ρ is the Spearman’s correlation coefficient, chosen due to its robustness against outliers and any normalization issues (44). To visually evaluate the differences in expression evolution, we constructed a tanglegram of the expression phylogenies of the two jaw tissues using the “dendextend” package (v1.19.0)(92) in R. Ancestral state reconstruction was conducted using the “phytools” package (v2.4.4) (93) in R.

### Phylogenetic Reconstruction (SNPs).

SNP calling on mapped mRNAseq reads was conducted using bcftools (94) across all samples. Filtered high quality SNPs were used to construct a concatenated alignment with the most common allele at each position for each individual. This alignment was used as input for RAxML (v8) (95) to construct a SNP phylogeny.

### GO Term Enrichment.

GO terms for *Oreochromis niloticus* were acquired in R via the biomaRt package (v2.46.1) (96) from ensembl (97). Gene set enrichment analysis was conducted using topGO (v2.36.0) (98) using the method weight to account for GO topology and Fisher’s exact test to correct for multiple testing for the enrichment analysis. We used our super-annotation to build the gene universe.

## Supplementary Material

Appendix 01 (PDF)

Dataset S01 (XLSX)

## Data Availability

All data have been deposited to the NCBI SRA BioProject (PRJNA640176) (99). All bioinformatics code can be found on GitHub (89).

## References

[1] P. Neige, Events of Increased Biodiversity (ISTE Press—Elsevier, 2015).

[2] D. Brawand , The genomic substrate for adaptive radiation in African cichlid fish. Nature **513**, 375–381 (2014). 10.1038/nature13726 PMC4353498 25186727

[3] M. King, A. C. Wilson, Evolution at two levels in humans and chimpanzees. Science **1979**, 107–116 (1975). 10.1126/science.1090005 1090005

[4] D. L. Stern, Perspective: Evolutionary developmental biology and the problem of variation. Evolution (N Y). **54**, 1079–1091 (2000). 10.1111/j.0014-3820.2000.tb00544.x 11005278

[5] P. Singh, E. P. Ahi, The importance of alternative splicing in adaptive evolution. Mol. Ecol. **31**, 1928–1938 (2022). 10.1111/mec.16377 35094439

[6] D. Berner, W. Salzburger, The genomics of organismal diversification illuminated by adaptive radiations. Trends Genet. **31**, 491–499 (2015). 10.1016/j.tig.2015.07.002 26259669

[7] M. Barrier, R. H. Robichaux, M. D. Purugganan, Accelerated regulatory gene evolution in an adaptive radiation. PNAS **98**, 10208–10213 (2001). 10.1073/pnas.181257698 PMC56940 11517318

[8] G. A. Wray, The evolutionary significance of cis-regulatory mutations. Nat. Rev. Genet. **8**, 206–216 (2007). 10.1038/nrg2063 17304246

[9] H. Keren, G. Lev-Maor, G. Ast, Alternative splicing and evolution: Diversification, exon definition and function. Nat. Rev. Genet. **11**, 345–355 (2010). 10.1038/nrg2776 20376054

[10] W. Gilbert, Why genes in pieces? Nature **271**, 501–501 (1978). 10.1038/271501a0 622185

[11] P. Singh, C. Börger, H. More, C. Sturmbauer, The role of alternative splicing and differential gene expression in cichlid adaptive radiation. Genome Biol. Evol. **9**, 2764–2781 (2017). 10.1093/gbe/evx204 PMC5737861 29036566

[12] M. Carruthers , Ecological speciation promoted by divergent regulation of functional genes within African cichlid fishes. Mol. Biol. Evol. **39**, msac251 (2022). 10.1093/molbev/msac251 PMC10101686 36376993

[13] Y. Terai, N. Morikawa, K. Kawakami, N. Okada, The complexity of alternative splicing of hagoromo mRNAs is increased in an explosively speciated lineage in East African cichlids. PNAS **100**, 12798–12803 (2003). 10.1073/pnas.2132833100 PMC240698 14569027

[14] E. J. Richards , A vertebrate adaptive radiation is assembled from an ancient and disjunct spatiotemporal landscape. Proc. Natl. Acad. Sci. U.S.A. **118**, e2011811118 (2021). 10.1073/pnas.2011811118 PMC8157919 33990463

[15] P. Singh, E. P. Ahi, C. Sturmbauer, Gene coexpression networks reveal molecular interactions underlying cichlid jaw modularity. BMC Ecol. Evol. **21**, 1–17 (2021). 10.1186/s12862-021-01787-9 PMC8061045 33888061

[16] O. Seehausen, Lake Victoria Rock Cichlids (Verduyn Cichlids, 1996).

[17] K. Yamaoka, “Trophic ecomorphology of Tanganyikan cichlids” in Fish Communities in Lake Tanganyika, N. M. Kawanabe, M. Hori, Ed. (Kyoto University Press, 1997), pp. 27–56.

[18] A. J. Conith, R. C. Albertson, The cichlid oral and pharyngeal jaws are evolutionarily and genetically coupled. Nat. Commun. **12**, 5477 (2021). 10.1038/s41467-021-25755-5 PMC8445992 34531386

[19] R. C. Albertson, T. D. Kocher, Genetic and developmental basis of cichlid trophic diversity. Heredity (Edinb.). **97**, 211–221 (2006). 10.1038/sj.hdy.6800864 16835594

[20] G. Fryer, T. D. Iles, The Cichlid Fishes of The Great Lakes of Afrika (T.H.F. Publikations Inc, Neptune City, NJ, 1972).

[21] P. H. Greenwood, Cichlid fishes of Lake Victoria, East Africa: the biology and evolution of a species flock. Bull. Brit. Mus. (Nat. Hist.) Zool. **Suppl. 6**, **1**–134 (1974).

[22] T. D. Kocher, J. A. Conroy, K. R. McKaye, J. R. Stauffer, Similar morphologies of cichlid fish in Lakes Tanganyika and Malawi are due to convergence. Mol. Phylogenet. Evol. **2**, 158–165 (1993). 10.1006/mpev.1993.1016 8025722

[23] K. A. Young, J. Snoeks, O. Seehausen, Morphological diversity and the roles of contingency, chance and determinism in African cichlid radiations. PLoS One **4**, e4740 (2009). 10.1371/journal.pone.0004740 PMC2648897 19270732

[24] K. F. Liem, Evolutionary strategies and morphological innovations: Cichlid pharyngeal jaws. Syst. Zool. **22**, 425–441 (1973).

[25] A. S. Roberts-Hugghis, C. M. Martinez, K. A. Corn, P. C Wainwright, A classic key innovation constrains oral jaw functional diversification in fishes. *Evol. Lett.* **9**, 24–40 (2025). 10.1093/evlett/qrae046 PMC11790220 39906576

[26] I. Irisarri , Phylogenomics uncovers early hybridization and adaptive loci shaping the radiation of Lake Tanganyika cichlid fishes. Nat. Commun. **9**, 3159 (2018). 10.1038/s41467-018-05479-9 PMC6082878 30089797

[27] P. Singh , Phylogenomics of trophically diverse cichlids disentangles processes driving adaptive radiation and repeated trophic transitions. Ecol. Evol. **12**, e9077 (2022). 10.1002/ece3.9077 PMC9288888 35866021

[28] W. Salzburger, T. Mack, E. Verheyen, A. Meyer, Out of Tanganyika: Genesis, explosive speciation, key-innovations and phylogeography of the haplochromine cichlid fishes. BMC Evol. Biol. **5**, 17 (2005). 10.1186/1471-2148-5-17 PMC554777 15723698

[29] S. Koblmüller , Age and spread of the haplochromine cichlid fishes in Africa. Mol. Phylogenet. Evol. **49**, 153–169 (2008). 10.1016/j.ympev.2008.05.045 18582582

[30] H. Svardal , Ancestral hybridization facilitated species diversification in the Lake Malawi cichlid fish adaptive radiation. Mol. Biol. Evol. **37**, 1100–1113 (2020). 10.1093/molbev/msz294 PMC7086168 31821500

[31] J. I. Meier , Cycles of fusion and fission enabled rapid parallel adaptive radiations in African cichlids. Science (1979) **381**, eade2833 (2023). 10.1126/science.ade2833 37769075

[32] H. Nakamura, M. Aibara, M. Nikaido, Ancient standing genetic variation facilitated the adaptive radiation of Lake Victoria cichlids. Genes Genet. Syst. **98**, 93–99 (2023). 10.1266/ggs.23-00024 37495512

[33] H. Nakamura , Genomic signatures for species-specific adaptation in Lake Victoria cichlids derived from large-scale standing genetic variation. Mol. Biol. Evol. **38**, 3111 (2021). 10.1093/molbev/msab084 PMC8321545 33744961

[34] Y. H. E. Loh , Origins of shared genetic variation in African cichlids. Mol. Biol. Evol. **30**, 906–917 (2013). 10.1093/molbev/mss326 PMC3603313 23275489

[35] M. A. Conte, W. J. Gammerdinger, K. L. Bartie, D. J. Penman, T. D. Kocher, A high quality assembly of the Nile Tilapia (*Oreochromis niloticus*) genome reveals the structure of two sex determination regions. BMC Genomics **18**, 1–19 (2017). 10.1186/s12864-017-3723-5 PMC5414186 28464822

[36] M. Pertea , Stringtie enables improved reconstruction of a transcriptome from RNA-seq reads. Nat. Biotechnol. **33**, 290–295 (2015). 10.1038/nbt.3122 PMC4643835 25690850

[37] M. I. Love, W. Huber, S. Anders, Moderated estimation of fold change and dispersion for RNA-seq data with DESeq2. Genome Biol. **15**, 550 (2014). 10.1186/s13059-014-0550-8 PMC4302049 25516281

[38] C. J. Wright, C. W. J. Smith, C. D. Jiggins, Alternative splicing as a source of phenotypic diversity. Nat. Rev. Genet. **23**, 697–710 (2022). 10.1038/s41576-022-00514-4 35821097

[39] E. T. Wang , Alternative isoform regulation in human tissue transcriptomes. Nature **456**, 470 (2008). 10.1038/nature07509 PMC2593745 18978772

[40] J. L. Trincado , SUPPA2: Fast, accurate, and uncertainty-aware differential splicing analysis across multiple conditions. Genome Biol. **19**, 1–11 (2018). 10.1186/s13059-018-1417-1 PMC5866513 29571299

[41] G. Ast, How did alternative splicing evolve? Nat. Rev. Genet. **5**, 773–782 (2004). 10.1038/nrg1451 15510168

[42] K. Garg, P. Green, Differing patterns of selection in alternative and constitutive splice sites. Genome Res. **17**, 1015 (2007). 10.1101/gr.6347907 PMC1899112 17556528

[43] N. L. Barbosa-Morais, M. Irimia, Q. Pan, H. Y. Xiong, S. Gueroussov, The evolutionary landscape of alternative splicing in vertebrate species. Science **1979**, 1587–1594 (2012). 10.1126/science.1230612 23258890

[44] D. Brawand , The evolution of gene expression levels in mammalian organs. Nature **478**, 343–348 (2011), 10.1038/nature10532. 22012392

[45] J. Chen , A quantitative framework for characterizing the evolutionary history of mammalian gene expression. Genome Res. **29**, 53–63 (2019). 10.1101/gr.237636.118 PMC6314168 30552105

[46] P. J. Wittkopp , Evolutionary changes in *cis* and *trans* gene regulation. Nature **430**, 85–88 (2004). 10.1038/nature02698 15229602

[47] D. Joyce , An extant cichlid fish radiation emerged in an extinct Pleistocene lake. Nature **435**, 90–95 (2005). 10.1038/nature03489 15875022

[48] K. J. Parsons, A. Trent Taylor, K. E. Powder, R. C. Albertson, Wnt signalling underlies the evolution of new phenotypes and craniofacial variability in Lake Malawi cichlids. Nat. Commun. **5**, 1–11 (2014). 10.1038/ncomms4629 PMC4238940 24699776

[49] S. Paine-Saunders, B. L. Viviano, J. Zupicich, W. C. Skarnes, S. Saunders, Glypican-3 controls cellular responses to Bmp4 in limb patterning and skeletal development. Dev. Biol. **225**, 179–187 (2000). 10.1006/dbio.2000.9831 10964473

[50] Y. Li, Q. Li, Y. Longs, Z. Cui, Lzts2 regulates embryonic cell movements and dorsoventral patterning through interaction with and export of nuclear β-catenin in zebrafish. J. Biol. Chem. **286**, 45116–45130 (2011). 10.1074/jbc.M111.267328 PMC3248021 22057270

[51] P. Masonick, A. Meyer, C. D. Hulsey, A kiss of deep homology: Partial convergence in the genomic basis of hypertrophied lips in cichlid fish and human cleft lip. Genome Biol. Evol. **15**, evad072 (2023). 10.1093/gbe/evad072 PMC10195091 37140021

[52] J. I. Meier , Ancient hybridization fuels rapid cichlid fish adaptive radiations. Nat. Commun. **8**, 14363 (2017). 10.1038/ncomms14363 PMC5309898 28186104

[53] K. E. Powder, R. C. Albertson, Cichlid fishes as a model to understand normal and clinical craniofacial variation. Dev. Biol. **415**, 338–346 (2016). 10.1016/j.ydbio.2015.12.018 PMC4914429 26719128

[54] H. Yu , Frizzled 1 and frizzled 2 genes function in palate, ventricular septum and neural tube closure: General implications for tissue fusion processes. Development **137**, 3707–3717 (2010). 10.1242/dev.052001 PMC2964100 20940229

[55] N. Karagic, R. F. Schneider, A. Meyer, C. D. Hulsey, A genomic cluster containing novel and conserved genes is associated with cichlid fish dental developmental convergence. Mol. Biol. Evol. **37**, 3165–3174 (2020). 10.1093/molbev/msaa153 32579214

[56] Y. Hu , A novel autosomal recessive GJA1 missense mutation linked to craniometaphyseal dysplasia. PLoS One **8**, e73576 (2013). 10.1371/journal.pone.0073576 PMC3741164 23951358

[57] A. Duenser , Developmental tinkering of gene regulation facilitated super rapid adaptive radiation. bioRxiv [Preprint] (2024). 10.1101/2024.01.24.577063 (Accessed 27 April 2026).

[58] J. Dalcq , RUNX3, EGR1 and SOX9B form a regulatory cascade required to modulate BMP-signaling during cranial cartilage development in zebrafish. PLoS One **7**, e50140 (2012). 10.1371/journal.pone.0050140 PMC3507947 23209659

[59] A. Busch, K. J. Hertel, Evolution of SR protein and hnRNP splicing regulatory factors. Wiley Interdiscip. Rev. RNA **3**, 1–12 (2012). 10.1002/wrna.100 PMC3235224 21898828

[60] C. E. Rodríguez-Ramírez , Molecular mechanisms of Eda-mediated adaptation to freshwater in threespine stickleback. Mol. Ecol. **00**, 1–19 (2023). 10.1111/mec.16989 PMC12288785 37194086

[61] A. M. Jakšić, C. Schlötterer, The interplay of temperature and genotype on patterns of alternative splicing in *Drosophila melanogaster*. Genetics **204**, 315–325 (2016). 10.1534/genetics.116.192310 PMC5012396 27440867

[62] A. Jacobs, K. R. Elmer, Alternative splicing and gene expression play contrasting roles in the parallel phenotypic evolution of a salmonid fish. Mol. Ecol. **30**, 4955–4969 (2021). 10.1111/mec.15817 PMC8653899 33502030

[63] M. Seppala , Gas1 is a modifier for holoprosencephaly and genetically interacts with sonic hedgehog. J. Clin. Invest. **117**, 1575–1584 (2007). 10.1172/JCI32032 PMC1868789 17525797

[64] G. G. Simpson, Tempo and Mode in Evolution (Columbia University Press, 1944).

[65] D. Schluter, The Ecology of Adaptive Radiation (Oxford University Press, Oxford, 2000).

[66] D. A. Marques, J. I. Meier, O. Seehausen, A combinatorial view on speciation and adaptive radiation. Trends Ecol. Evol. **34**, 531–544 (2019). 10.1016/j.tree.2019.02.008 30885412

[67] J. Peñalba , The role of hybridization in species formation and persistence. *Cold Spring Harb. Perspect. Biol*. **16**, a041445 (2024). 10.1101/cshperspect.a041445 PMC11610762 38438186

[68] A. El Taher , Gene expression dynamics during rapid organismal diversification in African cichlid fishes. Nat. Ecol. Evol. **5**, 243–250 (2021). 10.1038/s41559-020-01354-3 PMC7610457 33230257

[69] J. Draghi, C. B. Ogbunugafor, L. Zaman, T. L. Parsons, Relaxed selection can speed the evolution of complex adaptations. bioRxiv [Preprint] (2024). 10.1101/2024.07.09.602773 (Accessed 28 April 2026).

[70] M. L. Tress, F. Abascal, A. Valencia, Most alternative isoforms are not functionally important. Trends Biochem. Sci. **42**, 408–410 (2017). 10.1016/j.tibs.2017.04.002 PMC6449685 28483377

[71] J. Merkin, C. Russell, P. Chen, C. B. Burge, Evolutionary dynamics of gene and isoform regulation in mammalian tissues. Science **1979**, 1593–1599 (2012). 10.1126/science.1228186 PMC3568499 23258891

[72] A. K. Wanek, C. Sturmbauer, Form, function and phylogeny: Comparative morphometrics of Lake Tanganyika’s cichlid tribe Tropheini. Zool. Scr. **44**, 362–373 (2015). 10.1111/zsc.12110 PMC4949720 27478295

[73] C. Sturmbauer, U. Hainz, S. Baric, E. Verheyen, W. Salzburger, Evolution of the tribe Tropheini from Lake Tanganyika: Synchronized explosive speciation producing multiple evolutionary parallelism. Hydrobiologia **500**, 51–64 (2003).

[74] E. P. Ahi, Signalling pathways in trophic skeletal development and morphogenesis: Insights from studies on teleost fish. Dev. Biol. **420**, 11–31 (2016). 10.1016/j.ydbio.2016.10.003 27713057

[75] Á. J. Láruson, S. Yeaman, K. E. Lotterhos, The importance of genetic redundancy in evolution. Trends Ecol. Evol. **35**, 809–822 (2020), 10.1016/j.tree.2020.04.009. 32439075

[76] H. Dong , A systematic CRISPR screen reveals redundant and specific roles for Dscam1 isoform diversity in neuronal wiring. *PLoS Biol.* **12**, e3003383 (2023). 10.1371/journal.pbio.3002197 PMC10325099 37410725

[77] C. C. R. Smith , Genetics of alternative splicing evolution during sunflower domestication. PNAS **115**, 6768–6773 (2018). 10.1073/pnas.1803361115 PMC6042098 29891718

[78] C. E. Wagner, L. J. Harmon, O. Seehausen, Ecological opportunity and sexual selection together predict adaptive radiation. Nature **487**, 366–369 (2012), 10.1038/nature11144. 22722840

[79] K. Fujimura, N. Okada, Development of the embryo, larva and early juvenile of Nile tilapia *Oreochromis niloticus* (Pisces: Cichlidae). Developmental staging system. Dev. Growth Differ. **49**, 301–324 (2007). 10.1111/j.1440-169X.2007.00926.x 17501907

[80] K. Fujimura, N. Okada, Bone development in the jaw of Nile tilapia *Oreochromis niloticus* (Pisces: Cichlidae). Dev. Growth Differ. **50**, 339–355 (2008). 10.1111/j.1440-169X.2008.01032.x 18430028

[81] K. Fujimura, N. Okada, Shaping of the lower jaw bone during growth of Nile tilapia *Oreochromis niloticus* and a Lake Victoria cichlid *Haplochromis chilotes*: A geometric morphometric approach. Dev. Growth Differ. **50**, 653–663 (2008). 10.1111/j.1440-169X.2008.01063.x 18707606

[82] E. Tetrault, B. Aaronson, M. C. Gilbert, R. C. Albertson, Foraging-induced craniofacial plasticity is associated with an early, robust and dynamic transcriptional response. Proc. R. Soc. B: Biol. Sci. **291**, 20240215 (2024). 10.1098/rspb.2024.0215 PMC11040245 38654651

[83] P. Le Pabic, W. J. Cooper, T. F. Schilling, Developmental basis of phenotypic integration in two Lake Malawi cichlids. Evodevo **7**, 1–26 (2016). 10.1186/s13227-016-0040-z PMC4721197 26798449

[84] FastQC: A quality control tool for high throughput sequence data. Available at: www.bioinformatics.babraham.ac.uk/projects/fastqc.

[85] A. M. Bolger, M. Lohse, B. Usadel, Trimmomatic: A flexible trimmer for Illumina sequence data. Bioinformatics **30**, 2114–2120 (2014). 10.1093/bioinformatics/btu170 PMC4103590 24695404

[86] A. Dobin , STAR: Ultrafast universal RNA-seq aligner. Bioinformatics **29**, 15–21 (2013). 10.1093/bioinformatics/bts635 PMC3530905 23104886

[87] H. Li , The sequence alignment/map format and SAMtools. Bioinformatics **25**, 2078–2079 (2009). 10.1093/bioinformatics/btp352 PMC2723002 19505943

[88] G. Pertea, M. Pertea, GFF Utilities: GffRead and GffCompare [version 1; peer review: 3 approved]. F1000Res. **9**, 2 (2020). 10.12688/f1000research.23297.1 PMC7222033 32489650

[89] P. Singh, Transcriptomics pipeline for accurate gene and isoform analysis. Zenodo. 10.5281/zenodo.15393254. Deposited 13 May 2025.

[90] R Core Team, R: A language and environment for statistical (2021), https://www.R-project.org/.

[91] E. Paradis, K. Schliep, Ape 5.0: An environment for modern phylogenetics and evolutionary analyses in R. Bioinformatics **35**, 526–528 (2019). 10.1093/bioinformatics/bty633 30016406

[92] T. Galili, Dendextend: An R package for visualizing, adjusting, and comparing trees of hierarchical clustering. Bioinformatics **31**, 3718–3720 (2015), 10.1093/bioinformatics/btv428. PMC4817050 26209431

[93] L. J. Revell, Phytools: An R package for phylogenetic comparative biology (and other things). Methods Ecol. Evol. **3**, 217–223 (2012).

[94] P. Danecek , Twelve years of SAMtools and BCFtools. Gigascience **10**, giab008 (2021). 10.1093/gigascience/giab008 PMC7931819 33590861

[95] A. Stamatakis, RAxML version 8: A tool for phylogenetic analysis and post-analysis of large phylogenies. Bioinformatics **30**, 1312–1313 (2014). 10.1093/bioinformatics/btu033 PMC3998144 24451623

[96] D. Smedley , BioMart – biological queries made easy. BMC Genomics **10**, 22 (2009). 10.1186/1471-2164-10-22 PMC2649164 19144180

[97] D. R. Zerbino , Ensembl 2018. Nucleic Acids Res. **46**, D754–D761 (2018). 10.1093/nar/gkx1098 PMC5753206 29155950

[98] A. Alexa, J. Rahnenfuhrer, topGO: Enrichment analysis for gene ontology, R package version 2.62.0. https://bioconductor.org/packages/topGO. Accessed 28 April 2026.

[99] P. Singh. Cichlid species from Lake Victoria, Malawi and Tanganyika Raw sequence reads. NCBI. https://www.ncbi.nlm.nih.gov/sra/?term=PRJNA640176. Deposited 27 April 2026.

