# Supplementary material for: Ancestral splice variation is a key substrate for rapid diversification in African cichlids
Source: Proc Natl Acad Sci U S A. 2026 May 12;123(20):e2516477123. doi: 10.1073/pnas.2516477123 (PMC13187723; doi:10.1073/pnas.2516477123)
Supplement: Supplementary file 1 — Appendix 01 (PDF) [file pnas.2516477123.sapp.pdf]

Ancestral splice variation is a substrate for rapid diversification in African cichlids

Pooja Singh *et al* 2026 PNAS

Supplementary Information

Table of Contents

Ancestral splice variation is a substrate for rapid diversification in African cichlids 1

Table S1 Species information 3

Table S2 RNA mapping statistics 4

Figure S1 Spearman's rank correlation coefficient (ρ) of gene expression (GE) and percent spliced in (PSI) 10

Figure S2 Profiling of alternative splicing and gene expression in cichlid radiations. 12

Figure S3 Evolution of transcriptome-wide gene and isoform expression in cichlid radiations. 12

Figure S4 Transcriptomic variation in non-radiating and radiating cichlids: genes expressed. 13

Figure S5 Transcriptomic variation in non-radiating and radiating cichlids: number of isoforms per gene expressed. ... 14

Figure S6 Evolutionary dynamics of differentially spliced genes between pharyngeal jaws (PJ) of herbivores and carnivores from each radiation. 15

Figure S7 Gene Ontology enrichment of differentially spliced genes between herbivores and carnivores for oral (OJ) and pharyngeal jaws (PJ) 17

Figure S8 Gene expression divergence in pharyngeal jaws of herbivore and carnivore species. 17

Figure S9 Gene Ontology enrichment of differentially expressed genes between herbivores and carnivores. 18

Supplementary Materials and Methods: qPCR analysis 18

Figure S10 quantitative PCR (qPCR) validation of convergently expressed genes between herbivores and carnivores in all three lakes. 20

Figure S11 Development of external morphology and craniofacial bone and cartilage in Neochromis omnicaeruleus from hatching to developmental stage 26 (end of postembryonic development). 21

Figure S12 Dissection strategy of oral and pharyngeal jaws (OJ, PJ) of stage 26 larvae used for RNA extractions. 21

Supplementary Materials and Methods: Geometric morphometric analysis 22

Figure S13 Landmark template shown on a specimen of the Lake Victoria endemic Neochromis omnicaeruleus. 23

Figure S14 Principal Component analysis (PCA) of 17 species from our studyplotted with lake-specific symbols (square LM; triangle LT; circle LV) and colour codes for trophic niches (carnivore red and orange; herbivore greenish colours and the omnivore in grey). 23

Figure S15 Negative and positive deformation of extrapolated mandible half PC1(x-axis) and PC2 (y-axis) at dorsal view (top) and lateral view (bottom). All deformation grids were extrapolated by the factor three. 24

Supplementary Results and Discussion: Geometric morphometric analysis *24*

Table S3: Sampling list for geometric morphometric analyses. *25*

References *26*

Table S1 Species information

Abbreviation	species	Trophic niche
	**Lake Malawi**	
Ah	*Aulonocara hansbaenschi* "Red Flush"	carnivore (invertebrates)
Lt	*Labeotropheus trewavasae* "Red Top Thumbi West"	herbivore (algae)
Ptb	*Petrotilapia* sp. "Thick bars"	herbivore (algae)
Py	*Petrotilapia* sp. "Yellow chin Chewere"	herbivore (algae)
Sf	*Sciaenochromis fryeri*	carnivore (small fish, invertebrates)
Tt	*Tropheops tropheops* "Makokola"	(algae, plankton)
	**Lake Tanganyika**	
Ch	*Ctenochromis horei*	carnivore (small fish, invertebrates)
Gp	*Gnathochromis pfefferi*	carnivore (invertebrates)
Pf	*Petrochromis famula* "Crocodile Island"	herbivore (algae, phytoplankton)
Pp	*Petrochromis polyodon* "East Zambia"	herbivore (algae)
Sd	*Simochromis diagramma*	Herbivore (algae, phytoplankton)
Tm	*Tropheus moorii* "Nakaku"	Herbivore (algae, phytoplankton)
	**Lake Victoria**	
Gh	*Gaurochromis hiatus* 'Mwanza Gulf'	carnivore (invertebrate)
Ps	*Paralabidochromis sauvagei* (Haplochromis sp. 'rock kribensis')	omnivore (invertebrate, algae)
Ht	*Haplochromis thereuterion*	carnivore (invertebrate, zooplankton)
Ml	*Mbipia lutea* ' Makobe island'	herbivore (algae)
No	*Neochromis omnicaeruleus* "Makobe Island"	herbivore (algae)
Prp	*Prognathochromis perrieri*	carnivore (small fish)
	**Non-radiating riverine cichlids that also entered lakes**	
Aa	*Astatoreochromis alluaudi*	carnivore (invertebrates)

Ab	*Astatotilapia burtoni* "chipwa"	omnivore (algae, invertebrates, fish remains, opportunistic feeder)

Table S2 RNA mapping statistics

Category	Uniquely mapped	Mapped to multiple loci	Mapped to too many loci	Unmapped: too short	Unmapped: other	Uniquely mapped (%)
Aa.OJ.1	5115846	319640	37437	922391	96069	78.8
Aa.OJ.2	4130953	273830	70124	785922	221024	75.4
Aa.OJ.3	5563207	352615	28881	1071263	54447	78.7
Aa.OJ.4	22083577	1376678	124565	4003993	244859	79.3
Aa.OJ.5	6853276	433792	38157	1279754	77271	78.9
Aa.PJ.1	5261149	325804	85758	835943	287483	77.4
Aa.PJ.2	3728025	295212	173541	750399	680595	66.2
Aa.PJ.3	4317572	237349	25333	737273	54274	80.4
Aa.PJ.4	4182693	256788	28696	654894	62223	80.7
Aa.PJ.5	4410212	276925	27654	794798	60153	79.2
Ab.OJ.1	4188515	256459	25177	685525	60472	80.3
Ab.OJ.2	5488288	322164	27270	927459	54556	80.5
Ab.OJ.3	4402449	253917	22692	703783	49417	81
Ab.OJ.4	5953687	347155	28348	1094816	58370	79.6
Ab.OJ.5	4979488	276587	21549	914409	39865	79.9
Ab.PJ.1	2760915	154480	21365	421941	46655	81.1
Ab.PJ.2	6501582	379333	64527	958128	148148	80.7
Ab.PJ.3	4795157	259492	30924	645173	64343	82.7
Ab.PJ.4	3510209	189632	16834	504030	32326	82.5
Ab.PJ.5	3038370	179106	23621	486109	44500	80.6
Ah.OJ.1	5307986	320283	25064	1080552	42680	78.3
Ah.OJ.2	6405151	389208	31086	1335849	51726	78
Ah.OJ.3	7154864	451190	50059	1401315	107229	78.1
Ah.OJ.4	6004083	358058	31666	1182590	58022	78.6
Ah.OJ.5	4680805	271972	22633	896581	41372	79.2

Ah.PJ.1	4818647	291360	23631	886664	40000	79.5
Ah.PJ.2	4135397	261319	25389	781787	45679	78.8
Ah.PJ.3	5348725	316474	23864	1047911	42703	78.9
Ah.PJ.4	4520939	262270	23939	807678	45280	79.9
Ah.PJ.5	4168385	241142	25891	697131	50276	80.4
Ch.OJ.1	5002452	262198	26178	1056652	64798	78
Ch.OJ.2	2497732	125361	14636	449960	28025	80.2
Ch.OJ.3	3829827	205598	19696	839198	49452	77.5
Ch.OJ.4	4444873	236075	21130	798869	50519	80.1
Ch.OJ.5	4034325	206288	20026	705193	47113	80.5
Ch.PJ.1	3479115	170941	17532	568334	38031	81.4
Ch.PJ.2	5992763	304085	27964	1619671	58417	74.9
Ch.PJ.3	5463718	268809	22711	848127	51220	82.1
Ch.PJ.4	5498193	269773	24618	934110	63134	81
Ch.PJ.5	4588238	227873	18698	791605	45964	80.9
Gh.OJ.1	5852007	335325	32142	1040482	53377	80
Gh.OJ.2	3667558	205744	22582	781410	36761	77.8
Gh.OJ.3	7658391	426771	35351	1352469	60089	80.3
Gh.OJ.4	5957258	349157	27034	1130333	47316	79.3
Gh.OJ.5	7008441	408006	29622	1368109	47848	79.1
Gh.PJ.1	3867659	203217	22476	760651	35686	79.1
Gh.PJ.2	4719313	253932	30370	933675	47881	78.9
Gh.PJ.3	4460176	231912	25418	876301	38296	79.2
Gh.PJ.4	5352295	292364	23408	1074392	42080	78.9
Gh.PJ.5	4717261	257892	22559	983354	40948	78.3
Gp.OJ.1	6022120	306589	14912	1193898	42450	79.4
Gp.OJ.2	4757192	228283	14902	1189862	36755	76.4
Gp.OJ.3	4928780	237200	15344	1261352	38242	76.1
Gp.OJ.4	3924503	189202	14650	1011090	39366	75.8
Gp.OJ.5	5472749	289954	84381	1013191	283665	76.6
Gp.PJ.1	5001575	269485	18017	1051661	52423	78.2
Gp.PJ.2	4427633	218902	19156	977264	60473	77.6
Gp.PJ.3	3686019	178769	23781	885692	61399	76.2
Gp.PJ.4	4123711	217958	54869	970168	195814	74.1
Gp.PJ.5	2549939	129874	29799	443173	76231	79
Ht.OJ.1	6911297	424070	33186	1458875	61305	77.8

Ht.OJ.2	3897780	229901	42169	771468	66083	77.8
Ht.OJ.3	6590962	361908	23565	1227627	38750	80
Ht.OJ.4	4482925	228603	26133	864845	41747	79.4
Ht.OJ.5	7182555	418861	34804	1368159	58894	79.2
Ht.PJ.1	3208610	178816	13082	701154	22684	77.8
Ht.PJ.2	2307733	135142	30230	468787	57286	76.9
Ht.PJ.3	5839520	303298	23626	1179712	44350	79
Ht.PJ.4	2976068	152394	17593	552974	30223	79.8
Ht.PJ.5	3275466	174130	18598	600324	30733	79.9
Lt.OJ.1	5592178	298660	25073	1083286	50746	79.3
Lt.OJ.2	5466662	303939	22271	993950	38203	80.1
Lt.OJ.3	7169613	400601	30072	1440579	49111	78.9
Lt.OJ.4	6231704	353789	27106	1185504	47860	79.4
Lt.OJ.5	6542186	363235	28388	1177952	55510	80.1
Lt.PJ.1	3848024	191386	16838	768084	39401	79.1
Lt.PJ.2	4994867	269158	16761	934318	29978	80
Lt.PJ.3	6274784	344150	23467	1137165	47741	80.2
Lt.PJ.4	4111017	230908	15048	799248	25916	79.3
Lt.PJ.5	5088894	260944	19563	851570	44490	81.2
Ml.OJ.1	4779528	271544	21486	982068	37772	78.5
Ml.OJ.2	5585149	317824	24680	1134813	44057	78.6
Ml.OJ.3	4709910	272067	21670	976167	47603	78.1
Ml.OJ.4	4584617	266829	23372	880669	54025	78.9
Ml.OJ.5	4891895	283361	23039	963523	49698	78.8
Ml.PJ.1	5039454	267255	26124	1169256	49811	76.9
Ml.PJ.2	5216451	278160	27837	1050162	54993	78.7
Ml.PJ.3	3889142	224610	34629	727339	98504	78.2
Ml.PJ.4	5251767	298476	29451	944191	70551	79.6
Ml.PJ.5	4477837	258137	23336	833693	51929	79.3
No.OJ.1	5395709	370500	26167	1186153	41410	76.9
No.OJ.2	5619181	362078	32970	1078713	50007	78.7
No.OJ.3	5823554	352085	28237	1214977	50064	78
No.OJ.4	4473735	268227	22884	931205	40737	78
No.OJ.5	6132995	396430	28533	1294114	41058	77.7
No.PJ.1	5352739	339834	45726	1163403	115779	76.3
No.PJ.2	5997956	342225	35373	907526	56537	81.7

No.PJ.3	4080045	229890	42161	789728	104422	77.8
No.PJ.4	3945021	217824	35181	856809	71230	77
No.PJ.5	3183942	234399	119507	816096	314206	68.2
Pf.OJ.1	7947807	404248	29027	1394316	79816	80.6
Pf.OJ.2	5791279	292900	29938	1334139	63852	77.1
Pf.OJ.3	5161361	258603	24777	1126350	58339	77.9
Pf.OJ.4	4563584	238081	25578	1330626	52181	73.5
Pf.OJ.5	4791825	232390	26580	808562	65159	80.9
Pf.PJ.1	7035279	345254	28508	1277919	84201	80.2
Pf.PJ.2	3960377	201228	24972	849788	58079	77.7
Pf.PJ.3	6174510	300868	38198	1298869	93285	78.1
Pf.PJ.4	4795750	220939	19148	679422	46102	83.2
Pf.PJ.5	5575886	263517	26392	813940	66816	82.6
Pp.OJ.1	8314060	436690	25611	1611437	66925	79.5
Pp.OJ.2	4829686	250440	18508	1107668	37463	77.4
Pp.OJ.3	1519548	78655	13269	2173517	32834	39.8
Pp.OJ.4	4737067	244597	69304	756769	170960	79.2
Pp.OJ.5	5412469	298584	27446	935672	68760	80.3
Pp.PJ.1	5315694	259600	15636	1123677	37141	78.7
Pp.PJ.2	4682035	223223	17253	1075753	34994	77.6
Pp.PJ.3	5074270	321073	212345	1344232	648179	66.8
Pp.PJ.4	3516755	189054	130294	606269	298631	74.2
Pp.PJ.5	4258398	210799	20458	626323	48020	82.5
Prp.OJ.1	4714895	242922	31775	937027	50828	78.9
Prp.OJ.2	6487462	363540	37729	1367218	89312	77.7
Prp.OJ.3	6025899	337751	32587	1290339	63548	77.8
Prp.OJ.4	7116572	405090	40104	1410959	96119	78.5
Prp.OJ.5	7775110	409071	48075	1421183	114281	79.6
Prp.PJ.1	5080689	249289	43275	1001970	83982	78.7
Prp.PJ.2	4903357	249223	26562	1052028	55304	78
Prp.PJ.3	5502725	288357	38907	1118220	92950	78.2
Prp.PJ.4	3738386	200981	22490	704760	53341	79.2
Prp.PJ.5	3305380	185694	18144	644620	43227	78.8
Ps.OJ.1	6399897	372071	35014	1394940	69499	77.4
Ps.OJ.2	5709661	311782	42946	1214005	82455	77.6
Ps.OJ.3	4626788	251769	39102	871993	64375	79

Ps.OJ.4	5263877	280740	32167	975656	62177	79.6
Ps.OJ.5	5181286	312443	30209	1056379	59093	78
Ps.PJ.1	5740880	318404	43152	1209451	102270	77.4
Ps.PJ.2	4352284	229640	54282	801223	96833	78.6
Ps.PJ.3	6111323	324170	61472	1172280	135023	78.3
Ps.PJ.4	5319457	267421	74648	947634	137690	78.8
Ps.PJ.5	5048018	280401	52025	994187	114215	77.8
Ptb.OJ.1	4563367	269206	18527	1063636	31511	76.7
Ptb.OJ.2	7114957	419676	40571	1378785	81318	78.7
Ptb.OJ.3	5302622	319323	25183	1098392	44805	78.1
Ptb.OJ.4	5343635	318605	33327	1028340	67922	78.7
Ptb.OJ.5	5970463	357750	33511	1165518	64540	78.6
Ptb.PJ.1	3742805	212035	17935	852540	36475	77
Ptb.PJ.2	4834135	277458	31463	838624	73872	79.8
Ptb.PJ.3	4238648	252073	19920	939627	37871	77.2
Ptb.PJ.4	4388179	247138	31849	842065	69195	78.7
Ptb.PJ.5	4644086	269247	34640	766917	79356	80.1
Py.OJ.1	5347906	295047	25753	958661	52108	80.1
Py.OJ.2	6205819	349638	25199	1147214	45857	79.8
Py.OJ.3	5661376	306552	24603	1038562	52388	79.9
Py.OJ.4	4897704	263633	23936	938567	49366	79.3
Py.OJ.5	4524067	246387	25532	796194	61593	80
Py.PJ.1	5602097	301933	24513	942736	45650	81
Py.PJ.2	6906283	379801	28108	1130821	50976	81.3
Py.PJ.3	5609316	301372	25567	938159	54045	81
Py.PJ.4	3640462	194391	19267	552975	40483	81.9
Py.PJ.5	4516777	246730	23451	736712	47925	81.1
Sd.OJ.1	5539180	299107	34642	1048749	86928	79
Sd.OJ.2	5783309	286283	26056	1047359	56147	80.3
Sd.OJ.3	4732049	229358	17728	1062104	35849	77.9
Sd.OJ.4	4975049	251903	22510	833429	51552	81.1
Sd.OJ.5	5941520	294972	25440	1001098	65225	81.1
Sd.PJ.1	3772485	209931	51991	879914	113635	75
Sd.PJ.2	7119169	347200	62171	1455446	158161	77.9
Sd.PJ.3	6914250	328658	59628	1553180	144880	76.8
Sd.PJ.4	5696568	261873	25096	779123	53850	83.6

Sd.PJ.5	5086543	221518	23637	724132	53164	83.3
Sf.OJ.1	7143170	433689	30287	1463953	52903	78.3
Sf.OJ.2	7146958	448993	35039	1358082	58811	79
Sf.OJ.3	5516493	357074	27027	1148832	46124	77.7
Sf.OJ.4	6186658	386130	26044	1268508	40333	78.2
Sf.OJ.5	5820808	308921	30235	960451	62498	81
Sf.PJ.1	3776798	220861	14725	835601	25838	77.5
Sf.PJ.2	6219309	396525	33331	1162388	67773	78.9
Sf.PJ.3	4308855	261284	23294	804396	43510	79.2
Sf.PJ.4	5041048	316753	24023	955537	43376	79
Sf.PJ.5	4924204	275088	56812	858274	149728	78.6
Tm.OJ.1	8516062	434545	30617	1385654	66778	81.6
Tm.OJ.2	5596680	296955	27078	1244071	55597	77.5
Tm.OJ.3	5345382	280030	38934	1041742	85567	78.7
Tm.OJ.4	3757697	201681	31147	806765	67636	77.2
Tm.OJ.5	4229479	225236	33997	633138	87473	81.2
Tm.PJ.1	7627311	405288	27440	1403580	64795	80
Tm.PJ.2	4940304	263708	21555	979902	47495	79
Tm.PJ.3	3027989	214018	144718	767184	494718	65.1
Tm.PJ.4	3788748	212760	40825	789187	132028	76.3
Tm.PJ.5	3703931	209783	62288	533882	138465	79.7
Tt.OJ.1	5390289	291650	18175	1151114	36510	78.3
Tt.OJ.2	4700444	274178	19728	941133	33420	78.7
Tt.OJ.3	6832043	393398	27203	1332545	45741	79.2
Tt.OJ.4	3735283	219732	24905	704799	53997	78.8
Tt.OJ.5	5699037	323644	34887	998449	91483	79.7
Tt.PJ.1	4001285	239133	14062	873044	24723	77.7
Tt.PJ.2	4129526	243516	14865	968433	24749	76.7
Tt.PJ.3	5274110	293746	21493	979832	44297	79.7
Tt.PJ.4	4472595	269953	30501	816241	68444	79.1
Tt.PJ.5	5180742	288256	24341	979550	55508	79.4

\

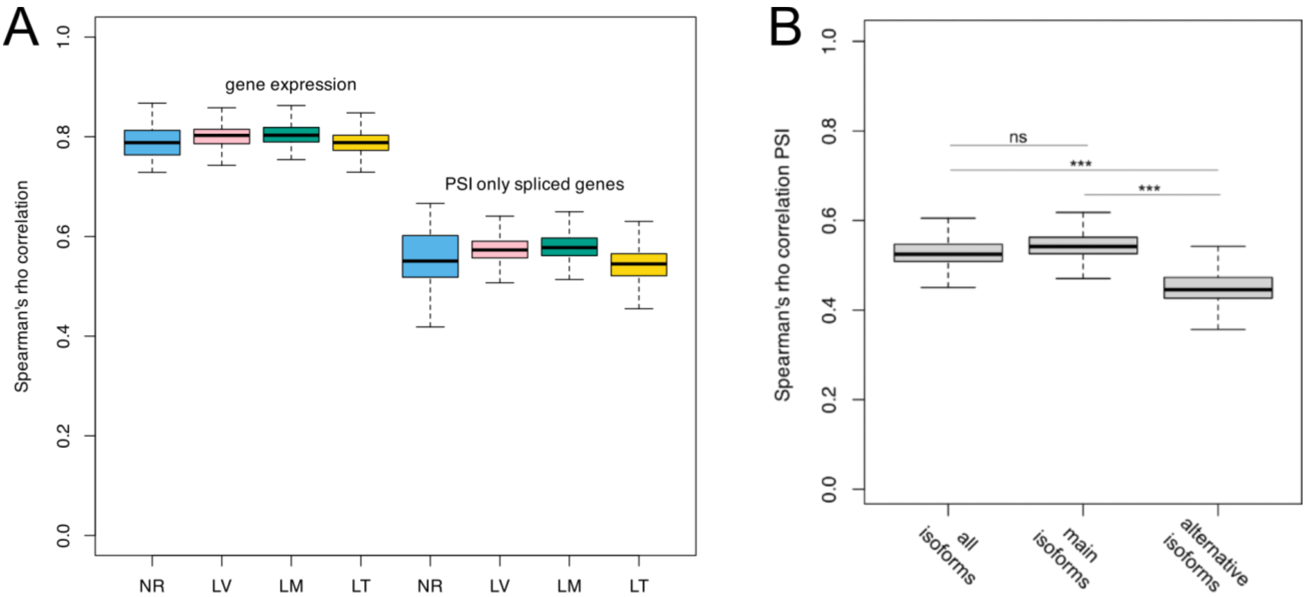

Figure S1 Spearman's rank correlation coefficient (ρ) of gene expression (GE) and percent spliced in (PSI)

(A) Boxplots of Spearman's ρ of PSI and GE calculated per lake (LT: Lake Tanganyika; LM:Lake Malawi; LV: Lake Victoria) as well as non-radiating species (NR). PSI is only calculated for genes with more than one isoform. All within lake and within non-radiating comparisons of gene expression rho versus PSI rho are significantly different (two-sided t-test $p < 0.001$). (B) Conservation of main and alternative isoforms in cichlid adaptive radiations. Distribution of Spearman's rank correlation coefficient (ρ) of isoform percent spliced in (PSI) between pairs of samples (n=200) for all genes with more than one isoform. Isoforms are categorised into main (constitutive) isoforms and alternative isoforms. Significant difference is calculated using two-sided t-test (*** = $p < 0.001$, ns = not significant).

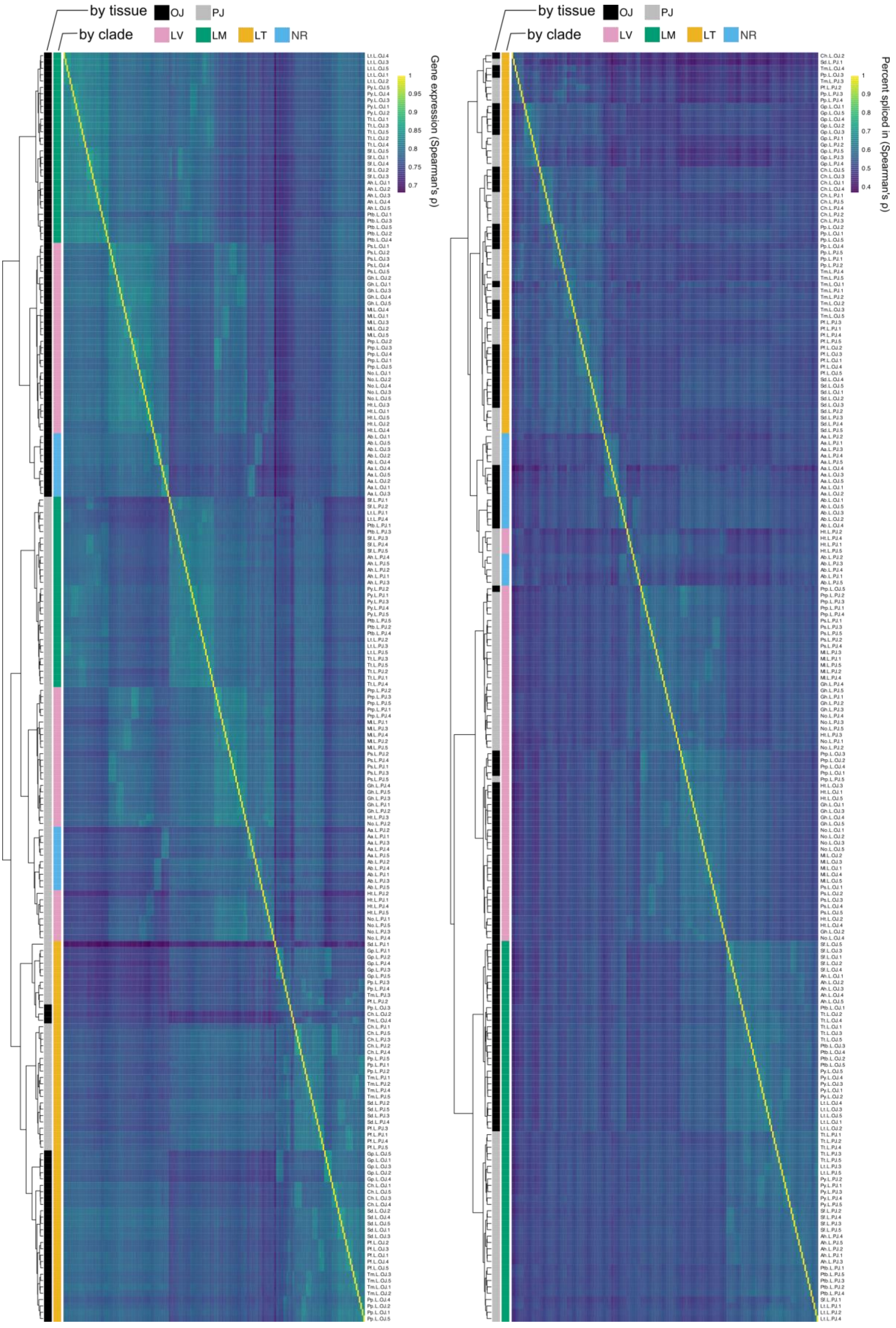

by tissue
OJ
PJ
by clade
LV
LM
LT
NR
Gene expression (Spearman's ρ)
1
0.95
0.9
0.85
0.8
0.75
0.7
by tissue
OJ
PJ
by clade
LV
LM
LT
NR
Percent spliced in (Spearman's ρ)
1
0.9
0.8
0.7
0.6
0.5
0.4

Figure S2 Profiling of alternative splicing and gene expression in cichlid radiations.

The same as main Figure 2 but with species names and tissue annotated next to rows. Hierarchical clustering of Spearman's rank correlation coefficient (ρ) of normalised gene expression and isoform percent spliced in (PSI) between pairs of samples (n=200). Sample clustering is represented as a tree on the left side of the heatmaps, with tissue and clade (radiations or nonradiating) of origin of species annotated in colours. *Paralabidochromis sauvagei* (Ps), *Mbipia lutea* (Ml), *Neochromis omnicaeruleus* (No), *Gaurochromis hiatus* (Gh), *Prognathochormis perrieri* (Pp), *Haplochromis thereuterion* (Ht), *Aulonocara hansbaenschi* (Ah), *Labeotropheus trewavasae* (Lt), *Petrotilapia* sp. 'Thick bars' (Ptb), *Petrotilapia* sp. 'Yellow chin Chewere' (Py), *Sciaenochromis fryeri* (Sf), *Tropheops tropheops* (Tt), *Ctenochromis horei* (Ch), *Gnathochromis pfefferi* (Gp), *Petrochromis famula* (Pf), *Petrochromis polyodon* (Pp), *Simochromis diagramma* (Sd), *Tropheus moorii* (Tm), *Astatoreochromis alluaudi* (Aa), *Astatotilapia burtoni* (Ab).

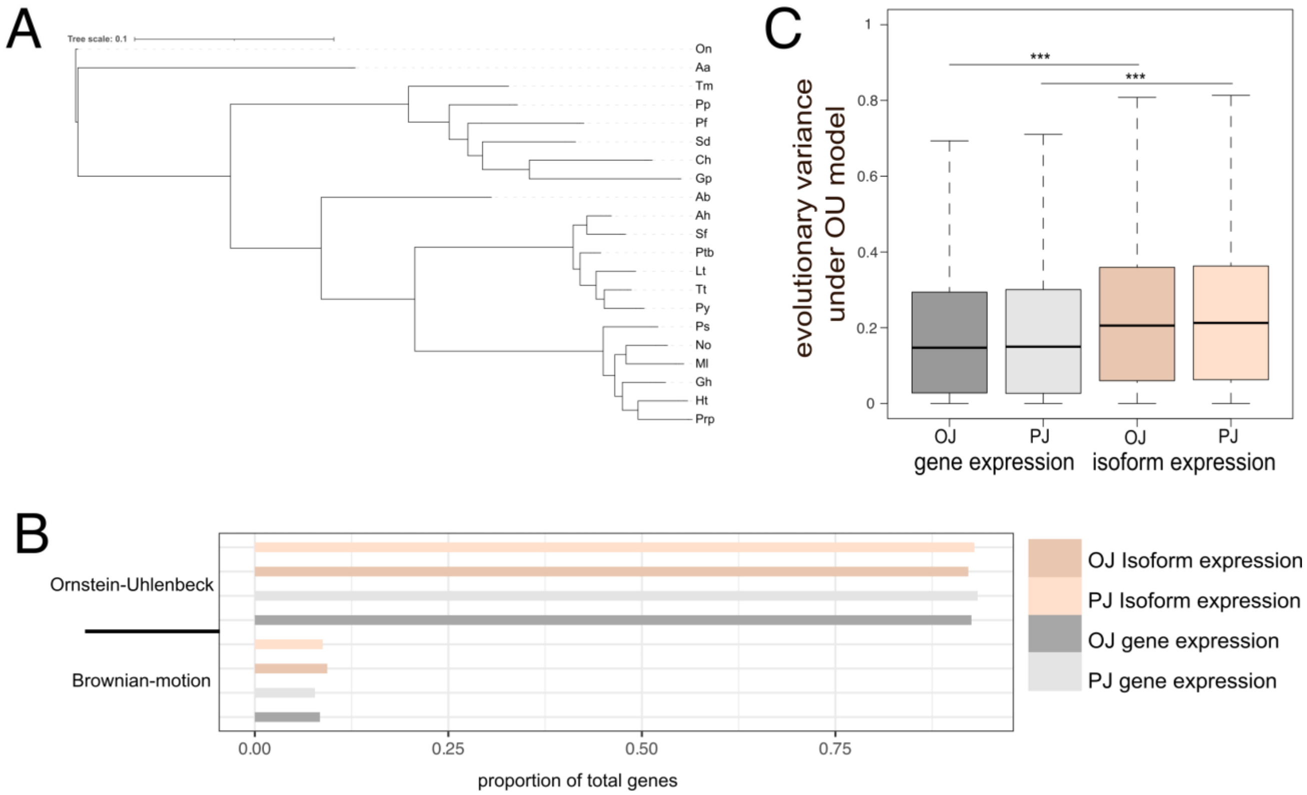

Figure S3 Evolution of transcriptome-wide gene and isoform expression in cichlid radiations.

(A) Phylogenetic reconstruction of all species in this study using transcriptomic SNPs. (B) Evolutionary variance in gene and isoform expression along the phylogeny under the Ornstein-Uhlenbeck (OU) model. (C) Number of genes and isoforms whose expression evolution along the phylogeny fits the Brownian motion (BM) or Ornstein-Uhlenbeck (OU) models.

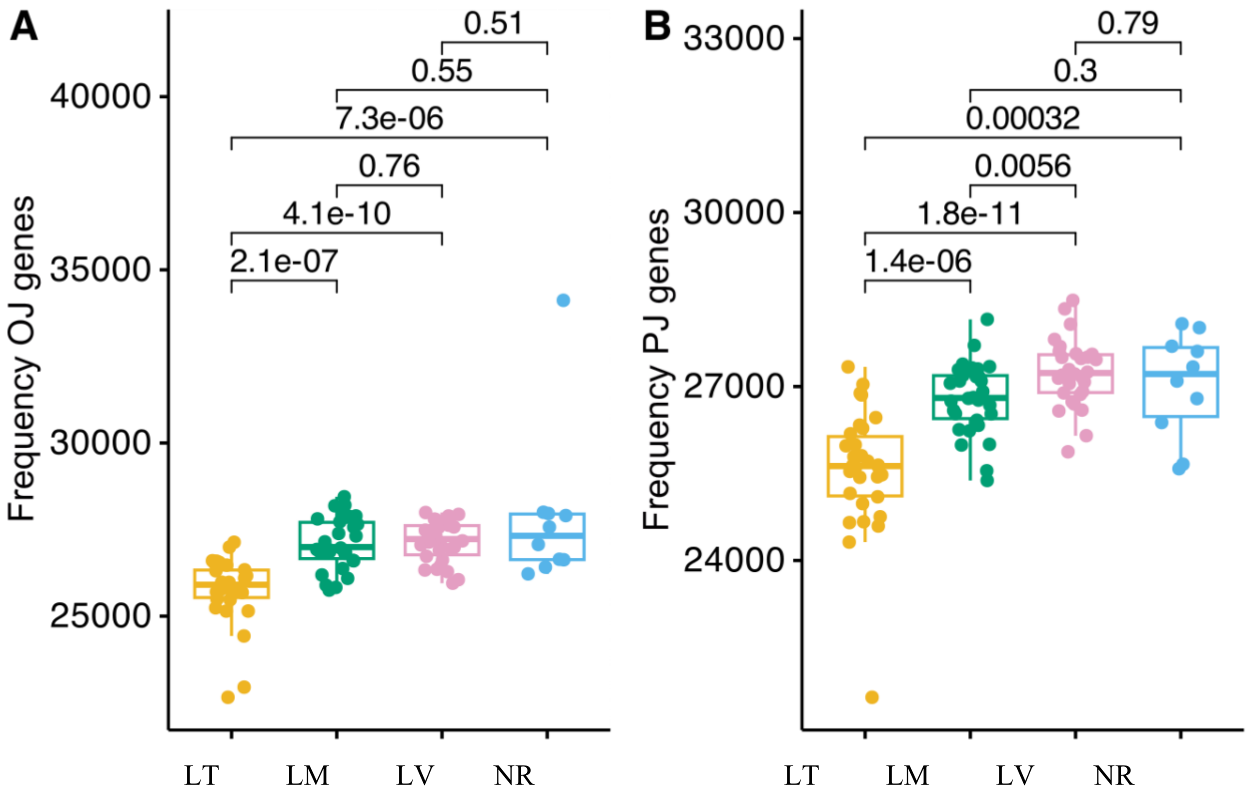

Figure S4 Transcriptomic variation in non-radiating and radiating cichlids: genes expressed. (A, B) The absolute number of expressed genes identified in the oral and pharyngeal jaw mRNA of all samples. *P*-values from Mann-Whitney U test between groups is shown. Groups: Lake Victoria (LV), Lake Malawi (LM) and Lake Tanganyika (LT) compared to non-radiating (NR)

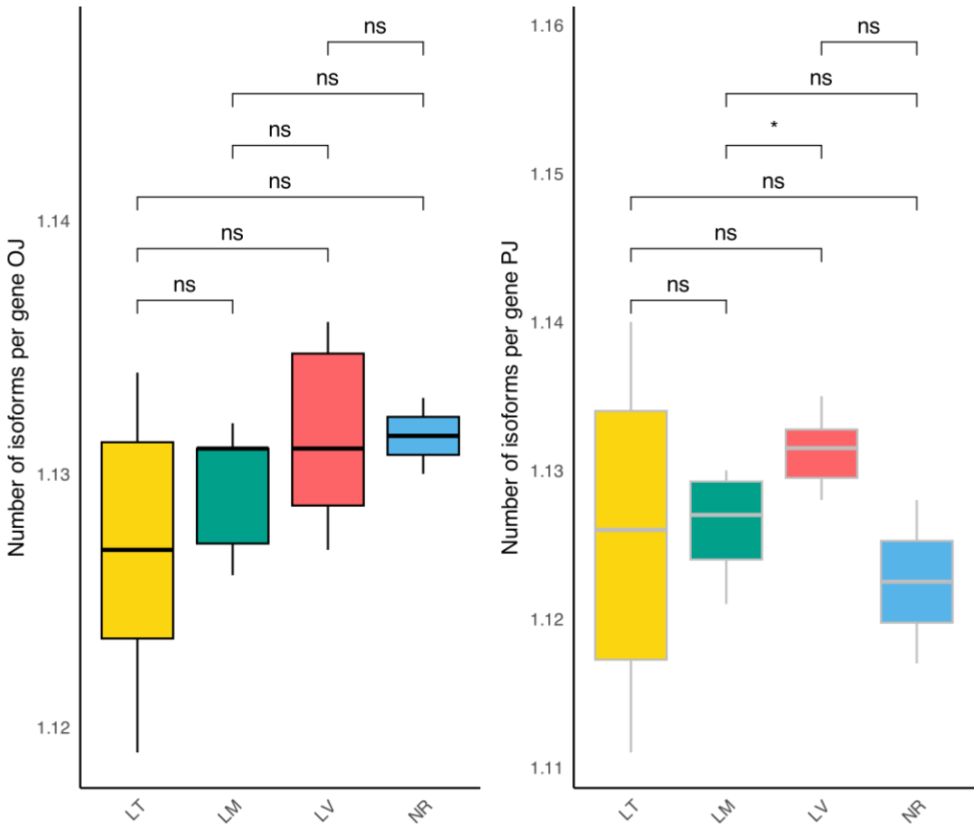

Figure S5 Transcriptomic variation in non-radiating and radiating cichlids: number of isoforms per gene expressed.

(A, B) The absolute number of expressed genes identified in the oral and pharyngeal jaw mRNA of all samples. Asterisks denote Mann-Whitney U test significance: $p < 0.05$* and NS: no significance. Groups: Lake Victoria (LV), Lake Malawi (LM) and Lake Tanganyika (LT) compared to non-radiating (NR)

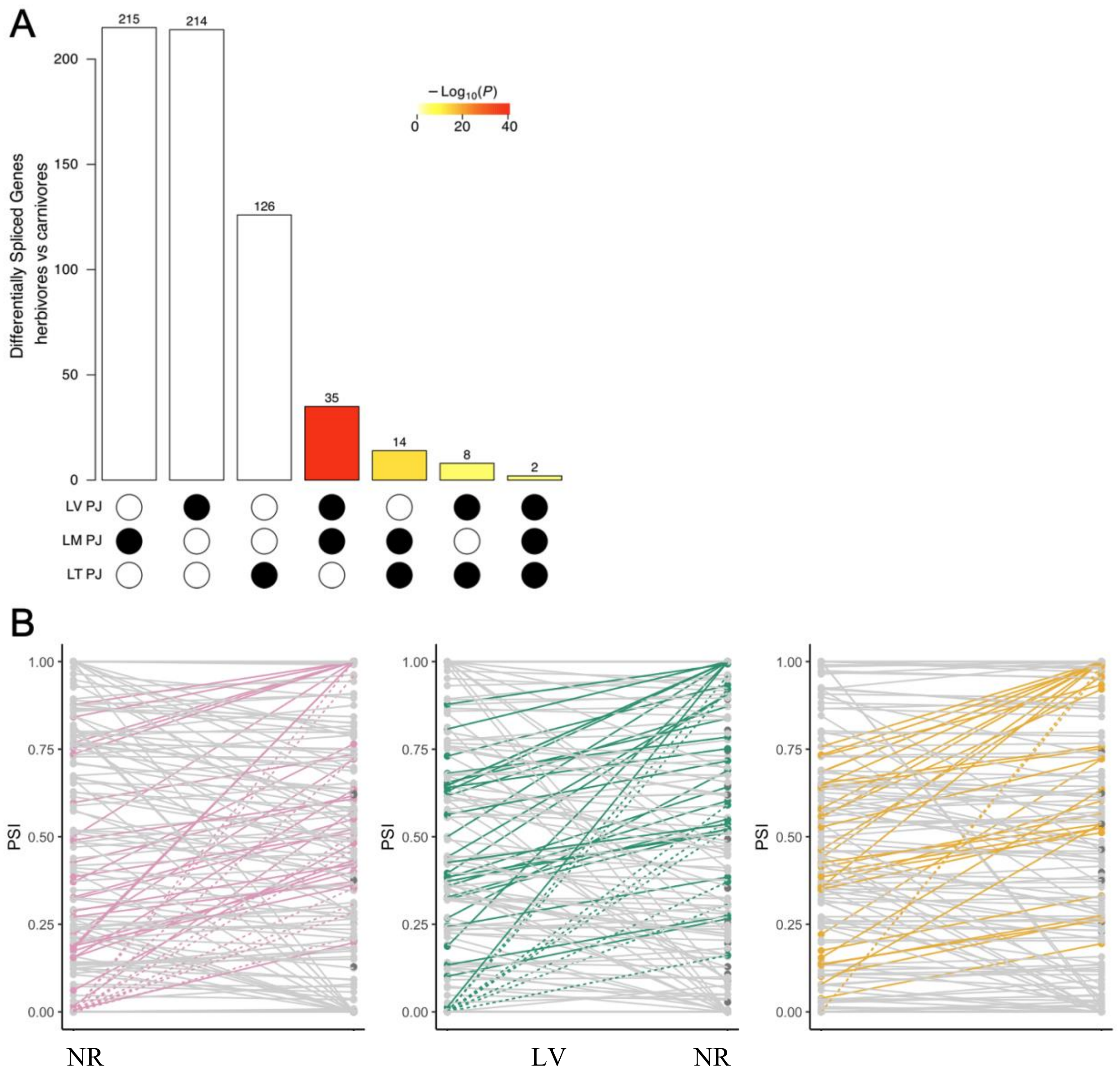

Figure S6 Evolutionary dynamics of differentially spliced genes between pharyngeal jaws (PJ) of herbivores and carnivores from each radiation.

(A) Number of significantly differentially spliced genes ($q < 0.05$) in PJ's of carnivores vs herbivores with significant overlaps shown (hypergeometric test *p* values are illustrated as heatmap). (B) Testing the hypothesis depicted in main Figure 3D in our transcriptome data from Lake Victoria (LV), Lake Malawi (LM) and Lake Tanganyika (LT) compared to non-radiating (NR) species that share a common ancestor with these radiations. Testing the hypothesis depicted in (D) in our transcriptome data from Lake Victoria radiation (LV), Lake Malawi radiation (LM) and Lake Tanganyika radiation (LT) compared to non-radiating species (NR). Mean PSI was calculated all individuals of all species in each group (NR, LV, LM, LT). All lines represent all isoforms of genes that were significantly differentially spliced between herbivores and carnivores within each radiation. Solid grey lines represent all isoforms that were expressed at lower or similar levels in the NR species and species from each radiation (PSI differences <= 0.2). Dotted grey lines represent all isoforms that were not expressed in NR species but were expressed species from each radiation (PSI

differences <= 0.2). Coloured solid lines indicate isoforms that were expressed at low levels in NR species but were higher expressed in radiating species (PSI differences > 0.2). Coloured dotted lines indicate isoforms that were not expressed in NR species but were expressed in radiating species (PSI differences > 0.2).

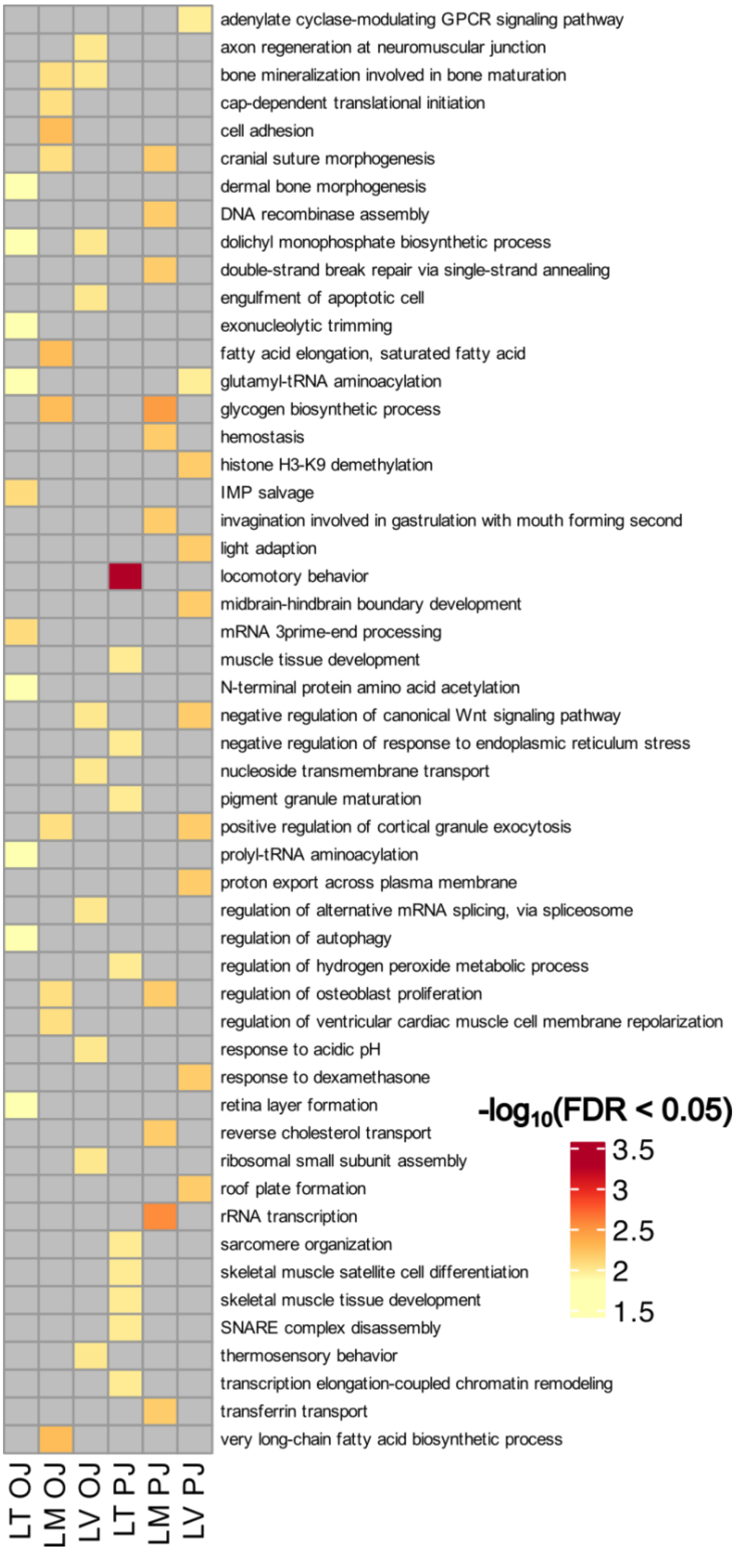

Figure S7 Gene Ontology enrichment of differentially spliced genes between herbivores and carnivores for oral (OJ) and pharyngeal jaws (PJ).

Only significant terms shown after correction for multiple testing (Fisher's exact test $q < 0.05$). LV: Lake Victoria, LM: Lake Malawi, LT: Lake Tanganyika, NR: non-radiating.

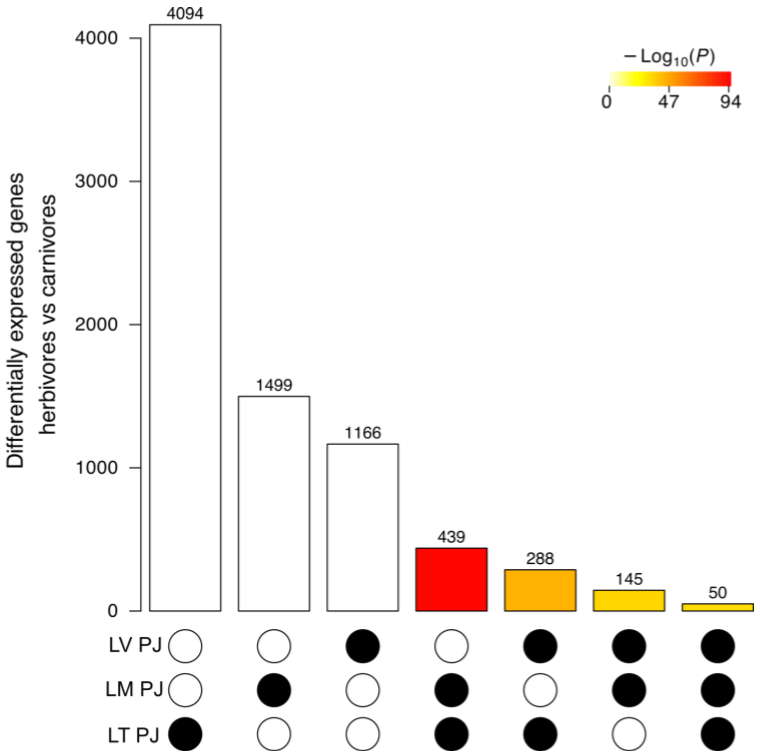

Figure S8 Gene expression divergence in pharyngeal jaws of herbivore and carnivore species.

Number of differentially expressed genes (DEGs, $q < 0.05$) between pharyngeal jaws (PJ) of herbivores and carnivores within Lake Victoria (LV), Lake Malawi (LM) and Lake Tanganyika (LT), with significantly greater overlaps than expected by chance are shown as a colour heatmap (hypergeometric test p values).

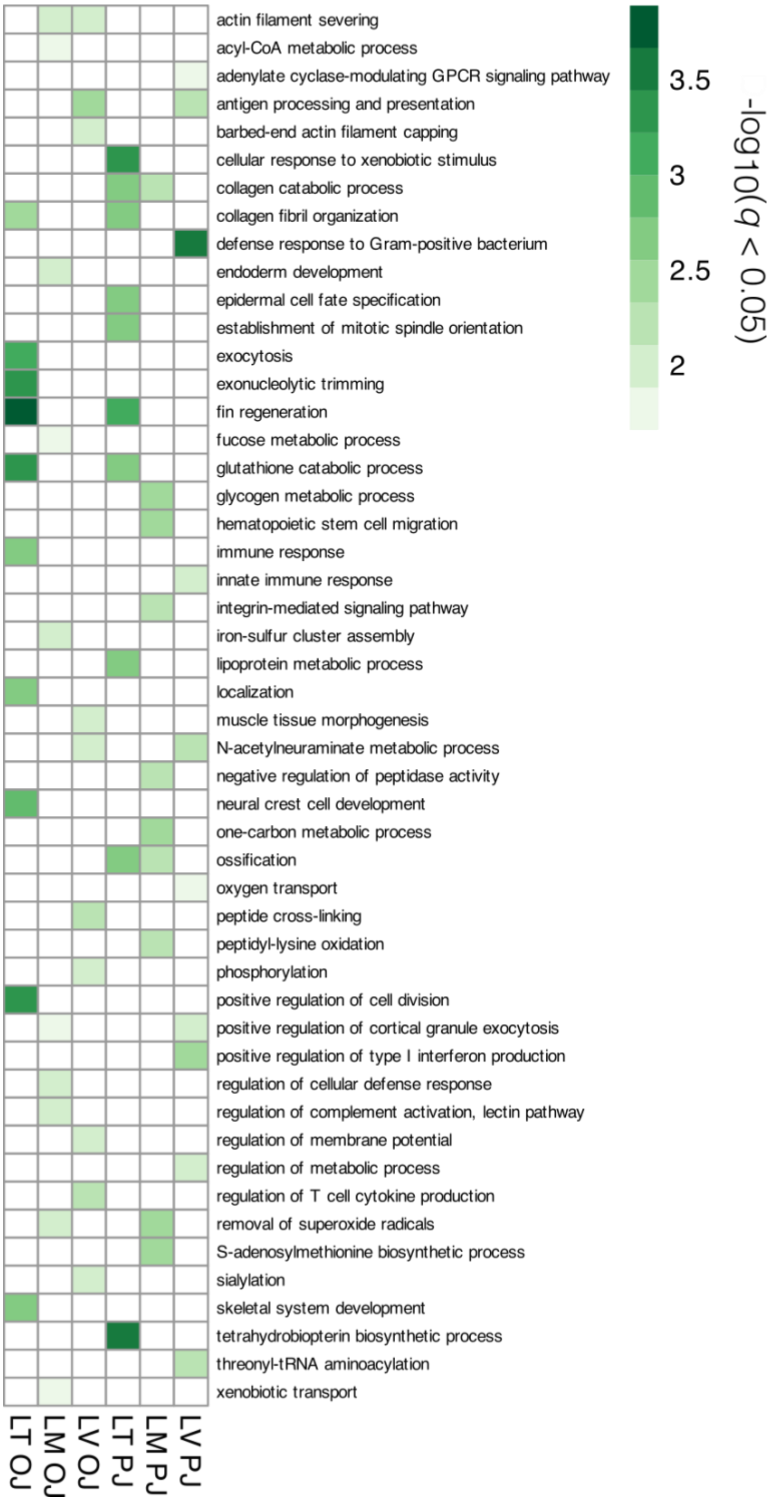

Figure S9 Gene Ontology enrichment of differentially expressed genes between herbivores and carnivores.

Only significant terms shown after correction for multiple testing (Fisher's exact test $q < 0.05$).

Supplementary Materials and Methods: qPCR analysis

From 4 replicates of RNA samples of each jaw, stage and species, we used 500ng of RNA to synthesize first strand cDNA using High Capacity cDNA Reverse Transcription kit (Applied Biosystems). The cDNA from each sample was diluted 1:10 times in nuclease-free water to be used for qPCR. We used rpl18 as a reference gene, which is validated as a stably expressed gene in both cichlid jaws across different trophic niches(1) to normalize our expression data for qPCR. The primers were designed at conserved sequence regions of the coding sequences from all species using CLC Genomic Workbench (v7.5 CLC Bio, Aarhus, Denmark) for alignment and delineation of exon boundaries using A. burtoni genome(2). Primer Express 3.0 (Applied Biosystems, CA, USA) and OligoAnalyzer 3.1 (Integrated DNA Technology) were used to design the primers as described in(1, 3). Maxima SYBR Green/ROX qPCR Master Mix (2X) (Thermo Fisher Scientific, Germany) was used to generate qPCR reactions. The amplification steps were conducted in 96 well-PCR plates on ABI 7500 real-time PCR System (Applied Biosystems). The primer efficiency calculation, qPCR program, plate set-up and a dissociation step were performed as described in(1, 3). To normalize the expression levels, the Cq value of rpl18 was used as normalization factor (Cq reference), and ΔCq of each target gene was calculated accordingly (ΔCq target = Cq target – Cq reference). Relative expression quantities (RQ) were calculated for the normalized values using $E^{-\Delta\Delta Cq}$ and then fold difference values were calculated by transformation of RQ values to logarithmic values in order to conduct further statistical analysis. The statistical expression differences were identified using ANOVA statistical tests, followed by Tukey's HSD post hoc tests.

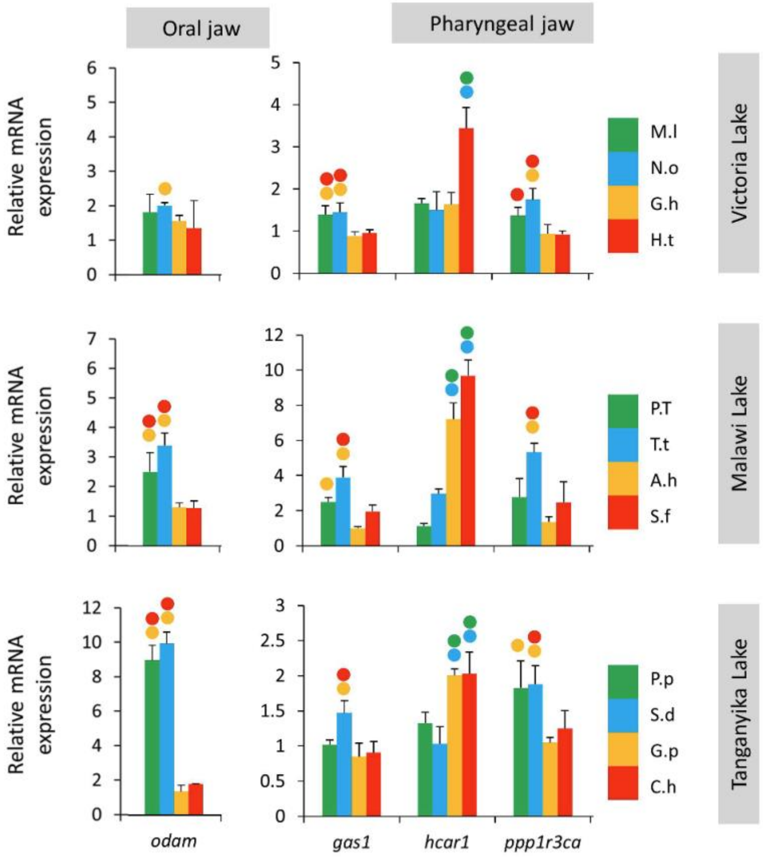

Figure S10 quantitative PCR (qPCR) validation of convergently expressed genes between herbivores and carnivores in all three lakes.

Blue/Green = herbivore species. Orange/Red – carnivore species. Species names are abbreviated. See Table 1 for full names. Filled circles above bars depict significantly different comparisons among groups.

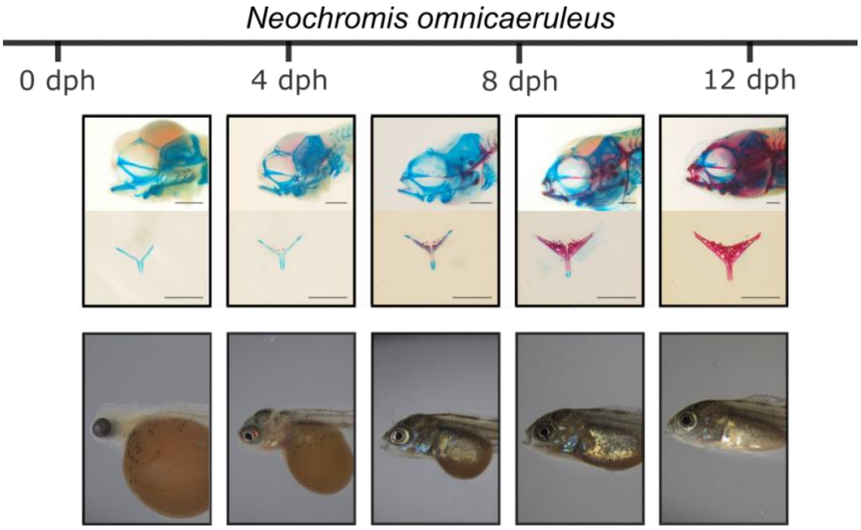

Figure S11 Development of external morphology and craniofacial bone and cartilage in *Neochromis omnicaeruleus* from hatching to developmental stage 26 (end of postembryonic development).

Top row: Alizarin red (bone) and Alcian blue (cartilage) stained images of *N. omnicaeruleus* sampled at regular intervals from the hatching to 12 days post hatching (dph). Images provided by B Dreo. Bottom row: external morphology of *N. omnicaeruleus* sampled at regular intervals over 12 dph, when egg yolk is absorbed into the body cavity and which signifies the end of the larval stage and the beginning of the juvenile stage, ad individuals leave the protection of the mother's buccal cavity to begin their independent life and feeding. Images provided by C Mlay.

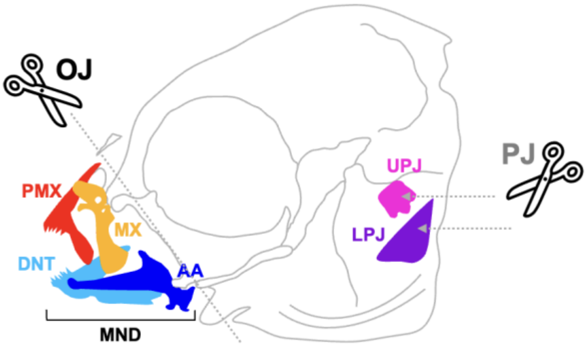

Figure S12 Dissection strategy of oral and pharyngeal jaws (OJ, PJ) of stage 26 larvae used for RNA extractions.

Oral jaw dissection includes the following bones: angulo-articular (AA), dentary (DNT), mandible (MND), maxilla (MX), pre-maxilla (PMX), as well as surrounding cartilage and muscle. Pharyngeal jaw dissection includes the upper pharyngeal jaw (UPJ) and the lower pharyngeal jaw (LPJ) as well as surrounding cartilage and muscle. For both OJ and PJ, we cleaned off as much surrounding tissue as possible under a stereomicroscope.

Supplementary Materials and Methods: Geometric morphometric analysis

We scanned the mandibles of 17 out of the 18 lacustrine study species, each represented by three adult male individuals, resulting in a total of 51 specimens analysed. According to our study design we bred and raised all specimens in our aquarium facility at the University of Graz under a standardised rearing and diet regime. We excluded the two riverine outgroups *Astatotilapia burtoni* and *Astatoreochromis alluaudi*, as well as the Lake Victoria endemic *Prognathochromis perrieri* for which only wild-caught specimens were available. The study species were classified in three trophic niches: omnivore, herbivore and carnivore (see supporting information Table S3). We used the SCANCO MEDICAL µCT 40 scanner to obtain the computer tomography scans and further reconstructed three-dimensional volumes by using the analytic software 3D Slicer (4). As studies in cichlids have shown that the craniofacial region comprises a major source of interspecific variation (5, 6), we focused on the mandible of the lower oral jaw. We defined a three-dimensional landmark set twelve homologous points which were uniformly well detectable on all study species (Fig. S13). Due to the oral symmetry of the used species, the left side was deemed sufficient for the morphometric analysis. For the descriptive termini of the precise landmark positions, we followed the classic paper (7). The complete landmark set is shown in Fig. S13 A to C, from different angles.

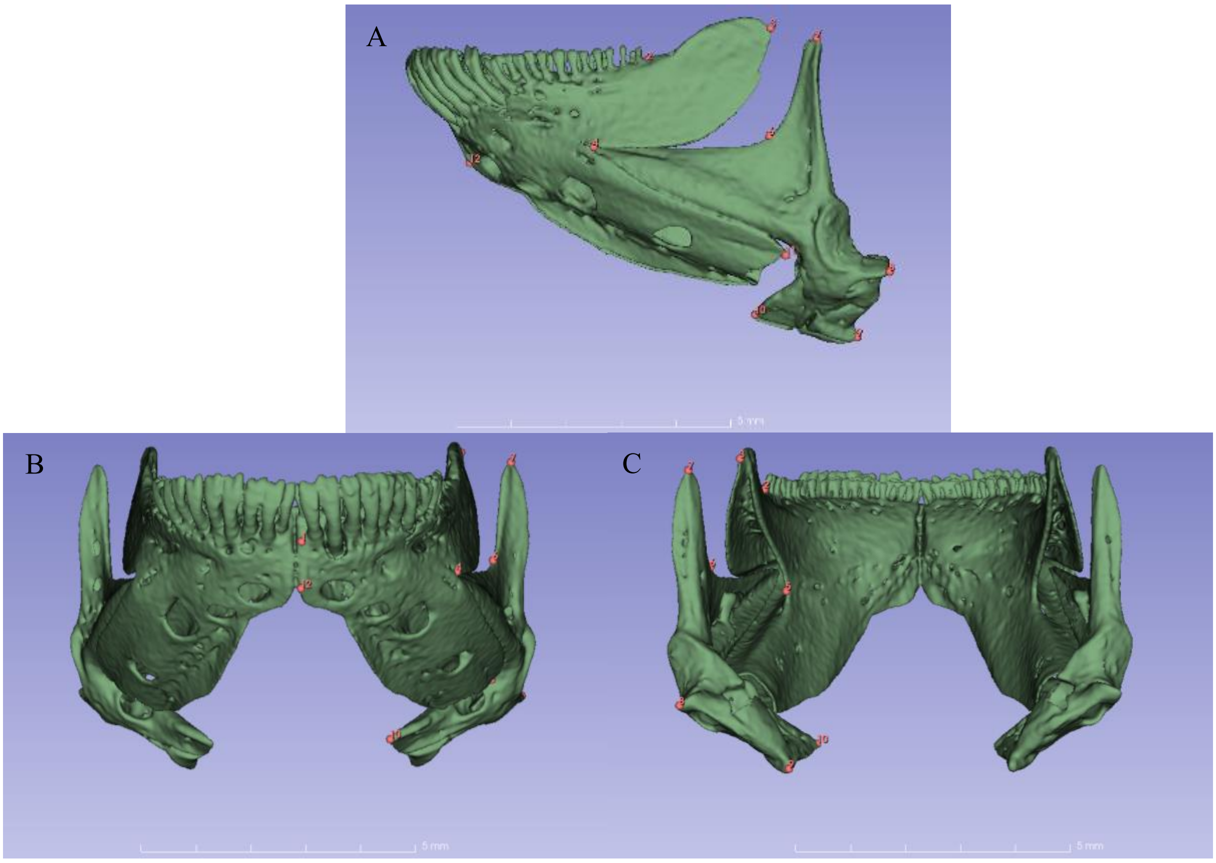

Figure S13 Landmark template shown on a specimen of the Lake Victoria endemic *Neochromis omnicaeruleus*.

(A) lateral view of the left side of the mandible. (B) anterior view. (C) posterior view. Number and position of landmarks: 1 - anterior dorsal teeth base, 2 - posterior teeth base, 3 - posterior tip of coronoid process, 4 - anterior tip of reentrant angle, 5 - dental fossa basin, 6 - articular web basin, 7 - post articular process tip, 8 - post articular process tip, 9 - posterior tip retroarticular process, 10 - anterior tip retroarticular process, 11 - posterior tip of ventral crest, 12 - anterior ventral tip of the dentary. The scale bar is 5 millimetres wide.

For the digitization of all 51 reconstructed mandible volumes and the generalized procrustes analysis (GPA) we used the integrated modules of 3D Slicer. We used the software package Past (8). We performed a PCA of all specimens (Fig. S14) and visualized shape differences between species with deformation grids of three-dimensional mandible volumes (Fig, S15) using 3D Slicer.

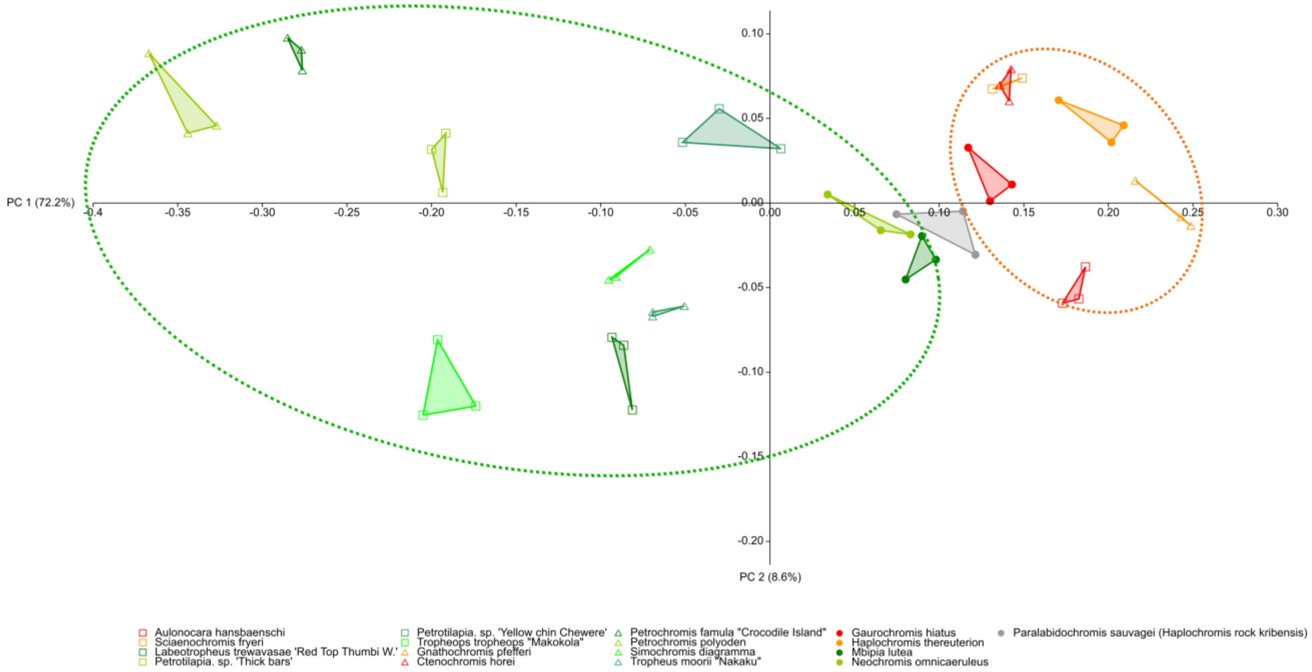

Figure S14 Principal Component analysis (PCA) of 17 species from our studyplotted with lake-specific symbols (square LM; triangle LT; circle LV) and colour codes for trophic niches (carnivore red and orange; herbivore greenish colours and the omnivore in grey).

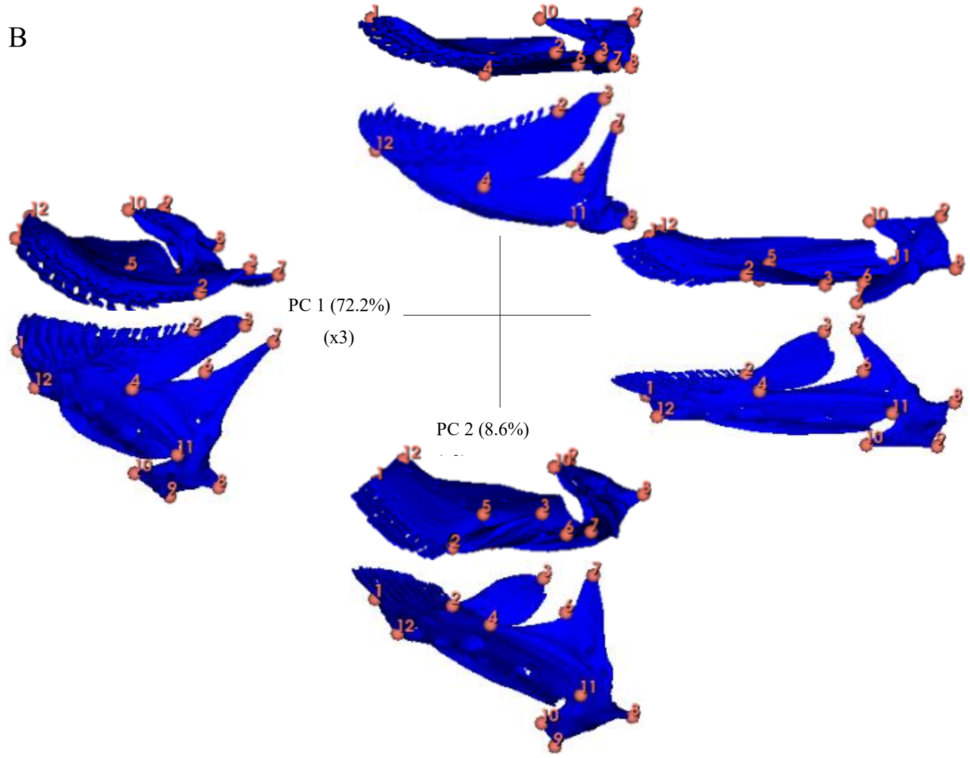

Figure S15 Negative and positive deformation of extrapolated mandible half PC1(x-axis) and PC2 (y-axis) at dorsal view (top) and lateral view (bottom). All deformation grids were extrapolated by the factor three.

Supplementary Results and Discussion: Geometric morphometric analysis

The first two principal components of the PCA explained total 80.8% of the variance: PC1 explained 72.2% of the variance, PC2 8.9% of the variance (Fig. S14). The separation of species regarding their trophic niche along the PC1-axies is distinctly visible for all lakes, with the herbivores clustering on the left side and the carnivore species clustering on the right side of the PC1 morphospace, with the omnivore *Paralabidochromis sauvagei* as intermediate. Within Lake Victoria the trend of trophic niche diverging along PC1 is visible, in line with the fact that Lake Victoria represents the youngest cichlid radiation between the three Great African Lakes. Lake Tanganyika as the oldest radiation shows the widest cluster distances among its species, and the Lake Malawi species comprise the second largest cluster range. Thus, Lake Victoria species show less interspecific mandible shape variation compared to Lakes Tanganika and Malawi, but species with similar trophic adaptations (herbivory vs carnivory) from each lake are diverging along parallel axes, consistent with previous findings (9).

The negative extrapolation along the PC1, shows a compressed mandible volume shape typical of herbivores (Fig. S15). In comparison the more elongated mandible volume along the positive extrapolation of PC1, similar to the shape of carnivores. Along the PC2-axis no separation between the species is possible, but the skewing of the articular process (Landmark 8 to Lankmark 10) is visible here.

Table S3: Sampling list for geometric morphometric analyses.

lake	diet	species	nr.	ID
LT	carnivore	*Gnathochromis pfefferi*	Gnpf_A_01	543
LT	carnivore	*Gnathochromis pfefferi*	Gnpf_A_02	544
LT	carnivore	*Gnathochromis pfefferi*	Gnpf_A_03	528
LT	herbivore	*Tropheus moorii "Nakaku"*	Trmo_A_01	460
LT	herbivore	*Tropheus moorii "Nakaku"*	Trmo_A_02	461
LT	herbivore	*Tropheus moorii "Nakaku"*	Trmo_A_03	462
LT	herbivore	*Simochromis diagramma*	Sidi_A_01	247
LT	herbivore	*Simochromis diagramma*	Sidi_A_02	254
LT	herbivore	*Simochromis diagramma*	Sidi_A_03	255
LT	herbivore	*Petrochromis famula "Crocodile Island"*	Pefa_A_01	426
LT	herbivore	*Petrochromis famula "Crocodile Island"*	Pefa_A_02	441
LT	herbivore	*Petrochromis famula "Crocodile Island"*	Pefa_A_03	442
LT	herbivore	*Petrochromis polyodon*	Pepo_A_01	469
LT	herbivore	*Petrochromis polyodon*	Pepo_A_02	534
LT	herbivore	*Petrochromis polyodon*	Pepo_A_03	538
LT	carnivore	*Ctenochromis horei*	Ctho_A_01	393
LT	carnivore	*Ctenochromis horei*	Ctho_A_02	394
LT	carnivore	*Ctenochromis horei*	Ctho_A_03	395
LV	omnivore	*Paralabidochromis sauvagei (Haplochromis rock kribensis)*	Pasa_A_01	451
LV	omnivore	*Paralabidochromis sauvagei (Haplochromis rock kribensis)*	Pasa_A_02	521
LV	omnivore	*Paralabidochromis sauvagei (Haplochromis rock kribensis)*	Pasa_A_03	522
LV	herbivore	*Mbipia lutea*	Mblu_A_01	525
LV	herbivore	*Mbipia lutea*	Mblu_A_02	526
LV	herbivore	*Mbipia lutea*	Mblu_A_03	527
LV	carnivore	*Gaurochromis hiatus*	Gahi_A_01	483
LV	carnivore	*Gaurochromis hiatus*	Gahi_A_02	484
LV	carnivore	*Gaurochromis hiatus*	Gahi_A_03	485
LV	carnivore	*Haplochromis thereuterion*	Hath_A_01	165
LV	carnivore	*Haplochromis thereuterion*	Hath_A_01	166
LV	carnivore	*Haplochromis thereuterion*	Hath_A_01	167
LV	herbivore	*Neochromis omnicaeruleus*	Neom_A_01	260
LV	herbivore	*Neochromis omnicaeruleus*	Neom_A_02	261
LV	herbivore	*Neochromis omnicaeruleus*	Neom_A_03	262
LM	herbivore	*Labeotropheus trewavasae "Red Top Thumbi West"*	Latr_A_01	447
LM	herbivore	*Labeotropheus trewavasae "Red Top Thumbi West"*	Latr_A_02	448

LM	herbivore	*Labeotropheus trewavasae "Red Top Thumbi West"*	Latr_A_03	449
LM	herbivore	*Tropheops tropheops "Makokola"*	Trtr_A_01	500
LM	herbivore	*Tropheops tropheops "Makokola"*	Trtr_A_02	509
LM	herbivore	*Tropheops tropheops "Makokola"*	Trtr_A_03	503
LM	herbivore	*Petrotilapia. sp. "Thick bars"*	Peth_A_01	505
LM	herbivore	*Petrotilapia. sp. "Thick bars"*	Peth_A_02	507
LM	herbivore	*Petrotilapia. sp. "Thick bars"*	Peth_A_03	537
LM	herbivore	*Petrotilapia. sp. "Yellow chin Chewere"*	Peye_A_01	371
LM	herbivore	*Petrotilapia. sp. "Yellow chin Chewere"*	Peye_A_02	373
LM	herbivore	*Petrotilapia. sp. "Yellow chin Chewere"*	Peye_A_03	374
LM	carnivore	*Sciaenochromis fryeri*	Scfr_A_01	154
LM	carnivore	*Sciaenochromis fryeri*	Scfr_A_02	155
LM	carnivore	*Sciaenochromis fryeri*	Scfr_A_03	156
LM	carnivore	*Aulonocara hansbaenschi*	Auha_A_01	490
LM	carnivore	*Aulonocara hansbaenschi*	Auha_A_02	491
LM	carnivore	*Aulonocara hansbaenschi*	Auha_A_03	492

References

Fedorov A, Beichel R, Kalpathy-Cramer J, Finet J, Fillion-Robin JC, Pujol S, Bauer C, Jennings D, Fennessy F, Sonka M, *et al.* 2012. 3D Slicer as an image computing platform for the Quantitative Imaging Network. *Magnetic Resonance Imaging* **30**: 1323–1341.

Hammer DAT, Ryan PD, Hammer Ø, Harper DAT. **2001**. Past: Paleontological Statistics Software Package for Education and Data Analysis. *Palaeontologia Electronica* **4**: 178.

Oijen MJP VAN, Barel CDN, Witte F. **1975**. The Shape of the Skeletal Elements in the Head of a Generalized Haplochromis Species: H. Elegans Trewavas 1933 (Pisces, Cichlidae). *Netherlands Journal of Zoology* **26**: 163–265.

Singh P, Börger C, More H, Sturmbauer C. **2017**. The role of alternative splicing and differential gene expression in cichlid adaptive radiation. *Genome Biology and Evolution* **9**: 2764–2781.

Wanek AK, Sturmbauer C. **2015**. Form, function and phylogeny: comparative morphometrics of Lake Tanganyika's cichlid tribe Tropheini. *Zoologica Scripta* **44**: 362–373.

Young KA, Snoeks J, Seehausen O. **2009**. Morphological Diversity and the Roles of Contingency, Chance and Determinism in African Cichlid Radiations (CR McClain, Ed.). *PLoS ONE* **4**: e4740.
